# The Polypill (Acetyl Salicylic Acid, Atorvastatin, and Ramipril) Paradigm Shift in Secondary Prevention: Global Expert Delphi Consensus

**DOI:** 10.5334/gh.1466

**Published:** 2025-09-24

**Authors:** Daniel Piñeiro, José Ramón González-Juanatey, Ana Abreu, Enrique Gómez Alvarez, Carlos Ponte-Negretti, Burkhard Weisser, Alexander Parkhomenko, Francisco Araújo, Alvaro Sosa-Liprandi

**Affiliations:** 1 School of Medicine, University of Buenos Aires, Argentina; 2 Hospital Clínico Universitario, Santiago de Compostela, Galicia, Spain; 3 Centro de Investigación Biomédica en Red Enfermedades Cardiovasculares (CIBERCV), Madrid, Spain; 4 Instituto de Investigación Sanitaria de Santiago (IDIS) Santiago de Compostela, Galicia, Spain; 5 Centro de Reabilitação Cardiovascular, S. Cardiologia, Centro Hospitalar Universitário Lisboa Norte e Faculdade Medicina Universidade de Lisboa (FMUL), CAML, Lisbon, Portugal; 6 Instituto de Medicina Preventiva e Instituto de Saúde Ambiental da FMUL, Lisbon, Portugal; 7 Centro Médico 20 de Noviembre, Mexico City, Mexico; 8 Instituto Médico La Floresta, Caracas, Venezuela; 9 Institute of Sports Science Christian Albrechts, University of Kiel, Kiel, Germany; 10 National Scientific Centre named after Strazhensku (former Insitute of Cardiology), Kyiv, Ukraine; 11 Hospital Lusíadas, Lisbon, Portugal; 12 Sanatorio Güemes, Buenos Aires, Argentina; 13 Inter-American Society of Cardiology, Mexico City, Mexico

**Keywords:** cardiovascular polypill, acute coronary syndrome treatment, secondary prevention, expert consensus, real-world practice

## Abstract

**Background:**

The SECURE trial demonstrated that the cardiovascular (CV)-polypill (acetylsalicylic acid [ASA] + atorvastatin + ramipril) reduces CV mortality by 33% in patients with acute myocardial infarction compared to standard care. The 2023 ACS ESC Guidelines recommend the polypill to improve outcomes and adherence.

**Objective:**

This study aims to establish a global consensus on the optimal use of the CV-polypill in secondary prevention.

**Methods:**

A two-round, modified Delphi method was used, featuring a 30-statement evidence-based questionnaire validated by eight renowned cardiologists. Fifty clinicians from 19 countries in Europe, Latin America, and Asia were invited to join the Delphi panel. Panelists ranked responses using a three-point Likert scale for agreement and importance. Consensus was defined as ≥80% agreement or rating statements ‘very important’ or ‘important’. Statements without consensus after the first round were refined with evidence and feedback in the second round. Remaining disagreements were resolved in a face-to-face meeting. Descriptive statistics were applied.

**Results:**

Response rate was 76% (round 1) and 74% (round 2); 82% were cardiologists, with 74% frequently recommending the CV-polypill. Consensus was achieved on 93.3% of statements. Research showing a 24% relative risk reduction in major adverse CV events over a median of 3 years with the CV-polypill post-acute myocardial infarction, compared to usual care, reached 97.4% agreement for clinical implementation, and a 100% consensus supported polypill use at hospital discharge or first follow-up visits; 81.1% agreed on a prompt initiation after patient stabilization. There was agreement on algorithms for initiating (97.3%), considering patient preferences (97.4%) to the polypill and its cost savings over usual care (89.5%).

**Conclusion:**

The Delphi consensus on real-world use of a CV polypill (ASA, atorvastatin, and ramipril) for secondary prevention post-acute coronary syndrome supports early initiation (within 8 days or at discharge). The findings provide a foundation to inform practice and policy, identifying priorities for further research.

## Graphical Abstract

**Figure:**
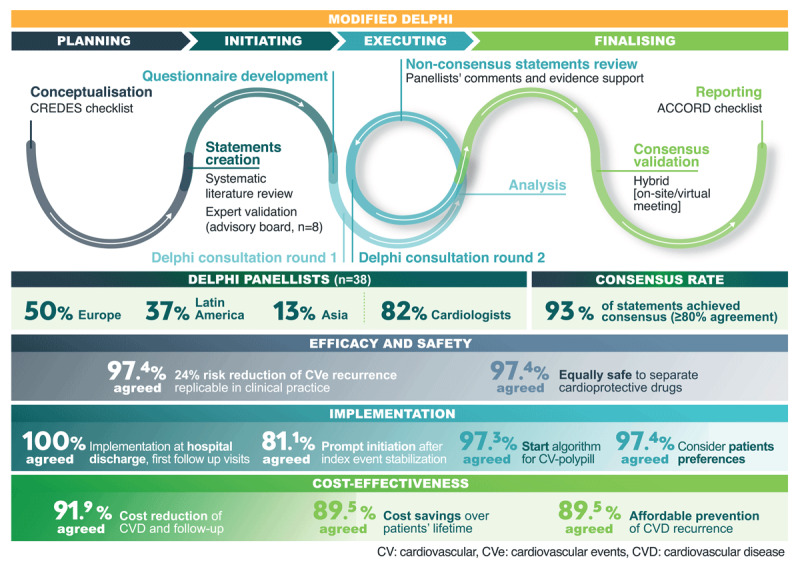

## Introduction

Cardiovascular disease (CVD) remains the leading cause of global adult mortality and disability (1). Secondary prevention population presents elevated cardiovascular (CV) event rates despite optimal medical treatment, ranging from 11.9 to 14.3 events per 100 patient-years (2,3). In the first 6 months post-CV event, there is a notable increase in events and deaths, often resulting in mortality (3), while over 5 years, 40% of patients may face death or recurrent CV events (3). Subsequent CV events tend to mirror the initial episode, particularly within cardiac or cerebrovascular clusters (2,3). These findings underscore a significant burden of CV events in real-world scenarios, emphasizing the ongoing challenge to optimize medical treatment (2).

Secondary prevention clinical practice guidelines consistently recommend antithrombotics, statins, and angiotensin-converting enzyme (ACE) inhibitors as the fundamental treatment for preventing recurrent CV events (4,5). These three cardioprotective drugs are combined in a single CV-polypill designed for patients with atherosclerotic cardiovascular diseases (ASCVDs) after a CV event (6). Treatment with this polypill has proven to reduce the incidence of recurrent major acute cardiovascular events (MACEs) (6).

The Secondary Prevention of Cardiovascular Disease in the Elderly (SECURE) trial demonstrated that the polypill strategy, containing ASA, atorvastatin, and ramipril, reduces CV mortality by 33% in patients with a previous acute myocardial infarction (AMI) over a 3-year (median) follow-up, compared to standard care (7). It stands as the only adequately powered secondary prevention trial, confirming the significant clinical benefits of the CV-polypill (ASA, atorvastatin, and ramipril) in secondary prevention (8).

Building on these results, the 2023 European Society of Cardiology (ESC) Guidelines for the management of acute coronary syndrome (ACS) supported the early use of a CV-polypill as an option to improve outcomes and adherence in secondary prevention post-ACS (5). Additionally, the World Health Organization (WHO) included for the first time a CV-polypill (ASA, atorvastatin, and ramipril) in its 2023 Essential Medicines List, aiming to improve accessibility and affordability of crucial cardiovascular medications worldwide (9). The World Heart Federation Roadmap for Secondary Prevention of Cardiovascular Disease in its 2023 update also included the use of a polypill to improve global availability and affordability of the necessary cardiovascular drugs (10). These hallmarks build on prior studies showcasing the polypill use to improve treatment adherence in real-world scenarios (11,12,13,14,15). Furthermore, extensive evidence supporting the CV-polypill, which contains ASA, atorvastatin, and ramipril, has facilitated its regulatory approval in 23 countries, marking a significant step toward global availability and accessibility.

Given the substantial clinical evidence of its efficacy and effectiveness (7,11,12,13,14,15), seeking consensus among experts was pivotal to assess the extent of shared recognition of this evidence. A real-world clinical meeting involving 30 participants from 18 countries and 4 expert discussants highlighted that, although guidelines and clinical trials support the CV polypill’s efficacy, key clinical questions remain unanswered in routine care, such as when to initiate treatment, how to transition from separate therapies, and how to determine appropriate dosing across patient profiles (16). This highlights a critical need for expert consensus to address the decision-making gap in real-world implementation.

The aim of this study was to achieve agreement among medical specialists on the optimal use of the CV polypill (ASA, atorvastatin, and ramipril) in post-ACS clinical practice. This core cardiovascular therapy is intended to prevent recurrent events and improve prognosis in secondary prevention patients in routine care.

## Methods

This study employed a modified two-rounds Delphi method to achieve consensus among expert panelists. The Guidance on Conducting and Reporting Delphi Studies (CREDESs) and the Accurate Consensus Reporting Document (ACOORD) checklists were applied to ensure compliance with methodological and reporting requirements (Supplementary Tables S1 and S2).

### Delphi consent round 1

The process involved a semi-structured questionnaire formulated in English, containing evidence-based statements derived from a systematic review of the literature (Supplementary Table S3). This search scope encompassed research on a polypill including lipid-lowering, antihypertensive, and antiplatelet drugs in adults with a history of ischemic cardiovascular or cerebrovascular disease. It considered clinical trials, observational studies, reviews, and guidelines worldwide, focusing on efficacy, endpoints, and various patient-related factors (Supplementary Table S4).

Initially, 58 publications were retrieved, and 17 were integrated into the questionnaire. Two additional hand-searched publications on medication management were added in Delphi round 2 based on panelists’ feedback (Supplementary Figure S1).

The questionnaire comprised 30 closed-ended statements in English, with panelists using a three-point Likert scale to express agreement on replicating the research findings of the polypill containing ASA, atorvastatin, and ramipril in clinical practice. Participants prioritized statements and ranked their importance to achieve consensus on relevance in everyday practice. The scale ranged from ‘agree’ to ‘disagree’, with prioritization from 1 to 5 and importance ranking as high, middle, or low.

The three-point Likert scale was chosen to ensure functionality, simplicity, and clarity for a diverse group of participants for whom English was a second language, in order to accurately gauge their perceptions.

The questionnaire was validated by the consensus Excellence Board (EB) and Executive Team (ET), who are the authors of this publication. Iterative revisions and two rounds of expert feedback were conducted to ensure clarity and relevance of the statements included in the questionnaire. Drafts were circulated among all EB and ET members, who reviewed the content independently and provided feedback. Revisions were made based on collective input, with final approval reached through consensus to ensure content validity and alignment with the objectives of the study.

The initial Delphi consensus round occurred from March 7 to April 20, 2023. The questionnaire was emailed to participants, who were encouraged to give feedback on statement wording, offer clarifications, or propose additional items relevant to the consensus topic that were not initially included.

### Delphi consent round 2

The second Delphi consensus round took place from May 31 to June 30, 2023, focusing on statements lacking consensus after round 1. Each statement underwent revision, incorporating participants’ suggestions and a thorough literature review for accuracy. Round 2 questionnaire included the original statement, agreement level from round 1, panelists’ comments, supporting literature arguments, and the revised statement (Supplementary Figure S2). The iterative process aimed to address unresolved statements and refine the consensus-seeking approach based on feedback and literature.

### Panelists

A total of 50 clinical experts with ≥5 years of experience in managing CVD and familiarity with the CV-polypill in their respective countries were invited to constitute the Delphi panel. Purposefully selected by the EB and ET, they represented expertise from 19 countries, ensuring an international perspective. The sample size was not intended to be representative at the country level and was not powered for subgroup analyses. The inclusion of experts from Armenia, Belgium, Chile, Costa Rica, Germany, Greece, Guatemala, Honduras, Italy, Jordan, Kazakhstan, Lebanon, Mexico, Portugal, the Republic of Belarus, the Dominican Republic, Serbia, Spain, and Ukraine aimed to gather insights into the CV-polypill strategy across diverse healthcare contexts. A confidentiality and collaboration agreement was signed prior to participation, specifying the voluntary nature of their involvement and that all responses would be anonymized through removal of any identifying information and reporting in aggregate form. Participants were reimbursed for their dedication according to standard professional rates.

### Consensus threshold

A consensus threshold of ≥80% was set to determine agreement or disagreement on statements during both consent rounds. An agreement meant ‘80% or more participants agree on the statement and its wording’. Statements falling below this threshold proceeded to round 2 for further discussion or rephrasing. In ranking scale questions, statements rated ‘very important’ or ‘important’ by ≥80% of participants after round 1 were retained, aligning with established Delphi practices. Disagreement was defined as ‘more than 50% of participants disagree or rank the statement as least important’. The thresholds for agreement (≥80%) and highest importance were set higher than those for discordance or lowest importance (>50%). Statements lacking consensus or needing rephrasing post-rounds were discussed in a face-to-face meeting with experts from consensus EB and ET, aiming to resolve disagreements.

## Statistical analysis

Returned questionnaires were analyzed using descriptive statistics, primarily frequencies and proportions, to summarize participants’ responses. Only statements with valid scores were included. Pearson correlation was used to assess consistency in responses across closely related statements, particularly those addressing efficacy, effectiveness, cost-effectiveness, and implementation of the CV-polypill, in cases where a unanimous consensus was not achieved. Missing responses were excluded from item-level analyses, and no imputation was performed. Inferential statistics were not applied, as the Delphi method is intended to explore expert agreement rather than test hypotheses. All analyses were conducted using Microsoft Excel.

### Role of the funding source

This study was funded by Ferrer International S.A. through an unrestricted grant, which had no influence on the development, data collection, analysis, or interpretation; nor on the writing of the report and manuscript, or the decision to submit the paper for publication.

## Results

### Response rate

A total of 38 (76%) and 37 (74%) panelists out of the initially invited 50 completed the questionnaire in rounds 1 and 2, respectively. Nine participants who originally agreed did not respond (20%), while 2 were unavailable (4%).

### Panelists

The Delphi panel consisted mostly of male participants (63%) with an average age of 55.3 (±9.6) years, primarily from Europe (50%), followed by Latin America (36.8%) and Asia (13.2%). The majority were cardiologists (82%) or internists (13%) with an average of 26.2 (±10.7) years in medical practice. A significant proportion (74%) of the panel frequently endorsed the CV-polypill treatment (ASA, atorvastatin, and ramipril), demonstrating their familiarity with its clinical use (Supplementary Table S5).

### Consensus overview

Twenty-three statements reached consensus in round 1. Seven statements proceeded to round 2 due to the lack of agreement (Supplementary Table S6). After both rounds, consensus was reached on 28 statements, resulting in a 93.3% consensus rate (28 out of 30). Two specific statements (#9c and #13) did not achieve consensus, necessitating face-to-face discussions with the experts (Supplementary Figure S3). The consensus was reached in accordance with the original plan with no deviations.

#### Objectives and recommendations for the cardiovascular polypill in everyday practice and hospital discharge

**Figure 1:**
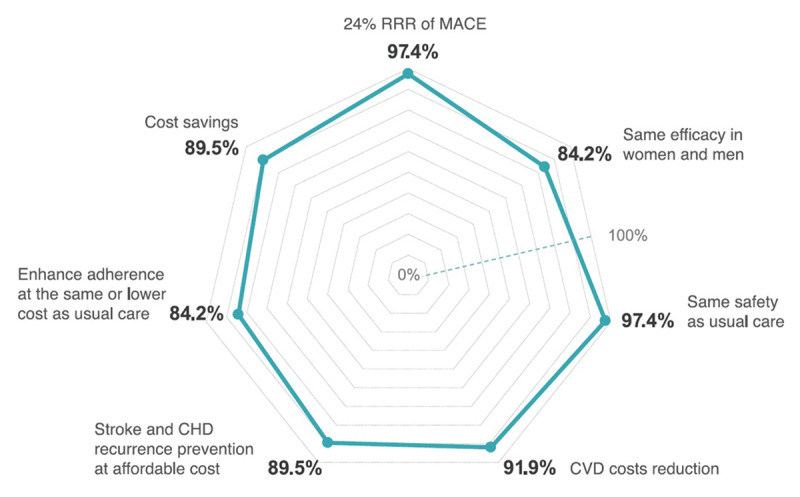
Radar chart: summary of agreement on aspects related to the efficacy, safety, and cost-effectiveness of the cardiovascular polypill. Cardiovascular polypill developed by the Centro Nacional de Investigaciones Cardiovascualres (CNIC), also known as CNIC-polypill. This polypill contains acetylsalicylic acid (100 mg), atorvastatin (20 or 40 mg), and ramipril (2.5, 5, or 10 mg) and available in 23 countries as Trinomia®, Sincronium®, or Iltria®. CHD: coronary heart disease, CVD: cardiovascular disease, MACE: major adverse cardiovascular event, RRR: relative risk reduction.

Among the Delphi panelists, 97.4% agreed that research findings, which demonstrate a 24% relative risk reduction in MACE over a median of 3 years post-AMI with a polypill (ASA, atorvastatin, and ramipril) compared to standard of care, can be replicated in real-world, routine clinical practice (Supplementary Table S7). This efficacy translates to both women and men alike (84.2% agreement). Panelists also affirmed its safety (97.4%), noting similar adverse events in magnitude and frequency compared to administering the cardioprotective drugs separately (Figure 1).

All panelists (100%) unanimously supported implementing a polypill as a treatment for preventing recurrence of events in post-ACS patients without contraindications either at the time of hospital discharge or during the initial follow-up visits (Supplementary Table S7). Eighty-one-point one percent (81.1%) agreed that initiating a polypill within eight days of an acute coronary event (replicating the median time to initiation on the SECURE trial) or immediately post-stabilization of symptoms is advisable, while 16.2% of panelists were neutral about this statement.

Among established ASCVD patients, 89.5% of panelists acknowledged that research findings demonstrating the effectiveness of the polypill (ASA, atorvastatin, ramipril) in helping them to achieve European guideline-recommended blood pressure and low-density lipoprotein cholesterol (LDL-c) target values after 2 years of treatment can be replicated in usual clinical practice (Supplementary Table S6).

**Figure 2:**
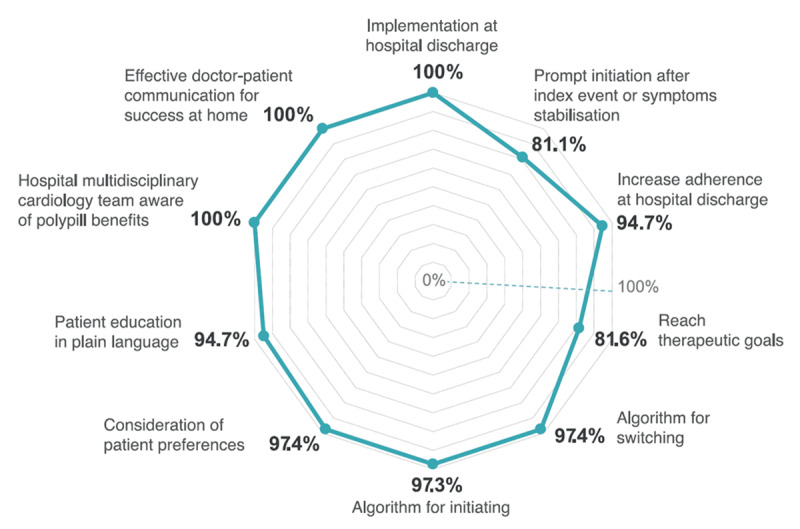
Radar chart: summary of agreement on aspects related to the implementation of the cardiovascular polypill at hospital discharge. Cardiovascular polypill developed by the Centro Nacional de Investigaciones Cardiovascualres (CNIC), also known as CNIC-polypill. This polypill contains acetylsalicylic acid (100 mg), atorvastatin (20 or 40 mg), and ramipril (2.5, 5, or 10 mg) and available in 23 countries as Trinomia®, Sincronium®, or Iltria®.

There was a strong agreement (97.3%) that published algorithms can be implemented when starting or transitioning to this polypill from multiple, separate cardioprotective drugs (Figure 2).

Ranking the importance of various objectives when recommending treatment with a polypill at the time of hospital discharge revealed that the most crucial and top-ranked objective (82.3%) was reducing the relative risk of cardiovascular death, AMI, or stroke through enhanced treatment adherence (94.7%). Controlling CVD risk factors and reducing polypharmacy were considered of slightly lesser importance (79.4%) by many participants compared to decreasing the rates of recurrent events and the risk of CVD death. Conversely, reducing treatment costs, enhancing safety, and increasing treatment satisfaction at discharge were of lower priority (Table 1).

**Table 1:** Percentage of panelists ranking of importance of objectives to be accomplished for recommending the cardiovascular polypill on hospital discharge.


	STATEMENT	RANK 1 (MOST IMPORTANT, SCORES 1 AND 2)*	RANK 2 (NEUTRAL, SCORE 3)*	RANK 3 (LEAST IMPORTANT, SCORES 4 AND 5)*

#21	What is the level of importance of the following objectives of the cardiovascular polypill treatment recommended for CHD secondary prevention patients on hospital discharge?			

	Please assign the level of importance to each of the objectives on a scale from 1 to 5. 1 = the most important and 5 = the least important.			

	To reduce the relative risk of acute myocardial infarction or stroke	82.3%	0.0%	17.7%

	To reduce the relative risk of cardiovascular death	82.3%	5.9%	11.8%

	To increase treatment adherence	82.3%	0.0%	17.6%

	To control CVD risk factors	79.4%	0.0%	20.6%

	To reduce polypharmacy/treatment burden	79.4%	2.9%	17.7%

	To increase treatment satisfaction	67.7%	11.8%	20.6%

	To reduce treatment cost	64.6%	26.5%	8.8%

	To increase safety	55.9%	23.5%	20.6%


*Note*: Neutral responses are shown for transparency. The ‘neutral’ column reflects participants who rated the statement as moderately important (score 3 in a 1 to 5 scale). A 0.0% indicates clear agreement (e.g., ‘to reduce risk of MI or stroke’); a higher percentage suggests varying stakeholder views and greater ambivalence about the importance of that statement (e.g., ‘to reduce treatment cost’). Consider all three columns together to assess the level of agreement. Cardiovascular polypill developed by the Centro Nacional de Investigaciones Cardiovascualres (CNIC), also known as CNIC-polypill. This polypill contains acetylsalicylic acid (100 mg), atorvastatin (20 or 40 mg), and ramipril (2.5, 5, or 10 mg) and available in 23 countries as Trinomia®, Sincronium®, or Iltria®. *Calculation based on 34 valid answers. CHD, coronary heart disease; CVD, cardiovascular disease.

According to participants, upon hospital discharge, recommending the CV-polypill should include various components: consideration of patients’ preferences (97.4%), patient education delivered in plain language (94.7%), discussion of the key elements of the treatment (92.1%), involvement of family or relatives in the discharge planning process (91.9%), and assessment of the explanation of the diagnosis and therapeutic strategy given by physicians and nurses (89.5%) (Figure 2 and Supplementary Table S7).

Furthermore, unanimous agreement (100%) was reached for the inclusion of advising on a healthy diet, smoking cessation, and providing guidance on daily physical activity (97.4%) as integral components of a comprehensive treatment plan when recommending the CV-polypill (Supplementary Table S7). There was a united consensus (100%) that all members of a hospital’s multidisciplinary cardiology team should be knowledgeable about the potential benefits of the polypill treatment in preventing the recurrence of CVD events.

#### Successful implementation of the cardiovascular polypill in the domiciliary setting

All participants (100%) unanimously ranked effective doctor-patient communication as the paramount factor for the success of the implementation of a polypill containing ASA, atorvastatin, and ramipril in an ambulatory setting following hospital discharge (Supplementary Table S7). The patient’s willingness to persist with medication (94.6%) and access to medications (88.6%) were also deemed of highest importance. Similarly, maintaining a satisfactory doctor–patient relationship (86.5%), the patient’s knowledge about the treatment (83.3%), and the ability to manage polypharmacy post-discharge (83.3%) were crucial determinants for a large segment of panelists. Other factors, such as patients’ medication adherence habits (81.1%) and social/family support (80%), were also important to many participants (Table 2). Patients’ financial capacity did not meet the consensus threshold (75%) as a key factor in the successful implementation of the CV-polypill in home care.

**Table 2:** Percentage of panelists ranking factors for the success of the cardiovascular polypill treatment after hospital discharge.


	STATEMENT	RANK 1 (MOST IMPORTANT, SCORES 1 AND 2)*	RANK 2 (NEUTRAL, SCORE 3)*	RANK 3 (LEAST IMPORTANT, SCORES 4 AND 5)*

#29	In your view, how important are the following factors for the success of the ‘at home’ cardiovascular polypill treatment after hospital discharge of secondary prevention patients with CHD? Please enter a number to indicate the importance of each factor on a scale from 1 to 5. 1 = the most important and 5 = the least important factor.			

	Effective doctor–patient communication	100.0%	0.0%	0.0%

	Willingness to persist on medication	94.6%	2.7%	2.7%

	Access to medications	88.6%	11.4%	0.0%

	Satisfactory doctor–patient relationship	86.5%	13.5%	0.0%

	Patient’s knowledge of the treatment	83.3%	11.1%	5.6%

	Patient capacity to manage polypharmacy post-discharge	83.3%	8.3%	8.3%

	Patient’s knowledge of the disease	82.9%	5.7%	11.5%

	Patient medication-taking habits	81.1%	13.5%	5.4%

	Social/family support	80.0%	11.4%	8.6%

	Financial capacity	75.0%	22.2%	2.8%


*Note*: Neutral responses are shown for transparency. The ‘neutral’ column reflects participants who rated the statement as moderately important (score 3 in a 1 to 5 scale). A 0.0% indicates clear agreement (e.g., ‘to reduce risk of MI or stroke’); a higher percentage suggests varying stakeholder views and greater ambivalence about the importance of that statement (e.g., ‘to reduce treatment cost’). Consider all three columns together to assess the level of agreement. Cardiovascular polypill developed by the Centro Nacional de Investigaciones Cardiovascualres (CNIC), also known as CNIC-polypill. This polypill contains acetylsalicylic acid (100 mg), atorvastatin (20 or 40 mg), and ramipril (2.5, 5, or 10 mg) and available in 23 countries as Trinomia®, Sincronium®, or Iltria®. *Calculation based on 37 valid answers. CHD, coronary heart disease.

### Cost-effectiveness of the cardiovascular polypill

Over 90% of Delphi panelists (91.9%) supported the idea that implementing the CV-polypill containing ASA, atorvastatin, and ramipril significantly reduces health care costs. Panelists agreed (89.5%) that this polypill effectively prevents recurrent MACE at a manageable cost to the healthcare system compared to standard cardioprotective pharmacological treatment. They (84.2%) also suggested that a polypill-based treatment might enhance coronary heart disease (CHD) outcomes at an equal or lower cost, contingent upon varied drug pricing and reimbursement policies across countries (Figure 1).

Additionally, 89.5% of participants agreed that the polypill (ASA, atorvastatin, and ramipril) can lead to cost savings over a patient’s lifetime. This cost reduction would be due to fewer CVD events, reduced hospitalizations, and decreased productivity loss compared to using individual cardioprotective drugs separately (89.5%). Moreover, 89.5% of the panelists highlighted that those patients on a polypill may require fewer CV-related prescriptions compared to those receiving similar cardioprotective drugs separately (Supplementary Table S6).

Consensus on the potential barrier of acquisition costs for patients, even assuming financial capacity (#13, Supplementary Table S8), was not achieved. Agreement level varied by regions: 60% of Asian, 64.7% of European, and a higher 85.7% of Latin American participants agreed on this statement (Supplementary Table S9).

### Patient profile and polypill treatment decision

When deciding on recommending the CV-polypill beyond ACS patients with prior events, there was 100% agreement on its efficacy and safety for patients with CHD, 94.7% agreement for patients with previous strokes, and 92.1% agreement for patients with peripheral artery disease (Supplementary Figure 4).

Sociodemographic factors had minimal impact on polypill recommendations (Supplementary Figure 5a). The medical background, particularly previous coronary ischemic events (94.6%), strokes (91.9%), multiple vascular diseases (89.2%), and peripheral artery disease (83.8%), played a crucial role in assessing suitability. However, there was no agreement on using this polypill containing ASA + atorvastatin + ramipril in patients with only risk factors such as hypertension, hypercholesterolemia, diabetes mellitus, metabolic syndrome, or hypertriglyceridemia (Supplementary Figure 5b).

Panelists emphasized patients’ treatment history, including adherence (97.3%), polypharmacy (97.3%), and previous persistence on treatment (91.9%), as significant factors in deciding on the adoption of the CV-polypill post-CHD event (Supplementary Figure 5c and Supplementary Table S7).

### Satisfaction with the cardiovascular polypill

#### Patient satisfaction

Unanimous agreement (100%) stressed simplified treatment as key for patient satisfaction with the CV-polypill. A significant majority (94.7%) emphasized convenience, while consensus (86.5%) highlighted patients valuing clinical benefits over side effects. No definitive agreement (78.4%) existed on whether preventing recurring MACE significantly influences satisfaction (#9c, Supplementary Table S8).

#### Physician satisfaction

Key factors contributing to physician satisfaction with the polypill containing ASA, atorvastatin, and ramipril include reduction in recurrence risk of CV events (89.7%), improved adherence (100%), achievement of blood pressure and LDL-cholesterol goals (86.8%) as recommended by clinical practice guidelines, and minimal adverse events (84.2%; Supplementary Table S7).

### Patient adherence to the cardiovascular polypill

Daily tablet count (80%) and medical staff explanation (71.4%) had the highest influence on patients’ adherence to the polypill for Delphi panelists (Supplementary Table S10).

### Consistent agreements on cardiovascular polypill implementation

Strong positive correlations (*r* ≥ 0.50; *p* < 0.001) emerged between expert agreement on initiating or transitioning to the CV-polypill and a notable 3-year reduction in MACE risks. This aligns with post-hospital discharge polypill utilization, emphasizing patient considerations (Supplementary Table S11).

## Discussion

The results of the current Delphi consensus reflect the perspectives of experienced physicians, primarily cardiologists, or internal medicine specialists, each with an average of over two decades of practice across Europe, Latin America, and Asia. These experts routinely prescribe a CV-polypill consisting of ASA, atorvastatin, and ramipril as a therapy to prevent recurrent events in secondary prevention.

Based on the strong consensus achieved, panelists assert that the outcomes observed in clinical studies translate into routine clinical practice. They acknowledge that, compared to standard care involving separate cardioprotective drugs, the polypill (ASA, atorvastatin, and ramipril) significantly reduces the relative risk of CV death by 33% in patients following an AMI over a median of 3 years (7). A meta-analysis, involving over 6000 CVD patients on a polypill-based treatment, revealed lower CV mortality, a reduced risk of recurrent events, and improved adherence compared to standard care, further supporting the potential prognostic benefits of the CV-polypill (17). Importantly, risk reduction is maintained while ensuring an equivalent level of safety. Pairing efficacy and safety results, it has been estimated that adverse events would warrant discontinuation of the cardiovascular polypill in only 1% to 2% of patients, with fatal side effects occurring in fewer than 1 in 10,000 users, depending on the precise formulation (18).

From a practical perspective, physicians prioritize reducing relative risks associated with CV death, AMI, or stroke over solely controlling individual cardiovascular risk factors when advocating for the polypill containing ASA, atorvastatin, and ramipril, aligning with the evidence derived from the SECURE trial (7). The CV health benefits observed in the SECURE trial may derive from a potential synergistic effect among the CV-polypill components on top of adherence enhancement. A pharmacodynamic study revealed significant reductions in LDL-C, total cholesterol, and triglycerides, that led to an additional 7% decrease in LDL-C levels in the polypill arm compared to atorvastatin alone (19). The authors argue that the polypill may be as effective as doubling the statin dose in lowering LDL-C levels (19).

Panelists emphasized the early adoption of a comprehensive approach to treating CV diseases, favoring it over targeting individual CVD risk factors (6). The consensus underscores the belief that the treatment with a polypill containing ASA, atorvastatin, and ramipril should be promptly initiated or switched to optimize CV outcomes, provided there are no contraindications and CVD symptoms are adequately controlled, ideally at the point of hospital discharge. Nevertheless, there appears to be uncertainty regarding timing among approximately 10% of panelists, who maintain a neutral position on this issue. In alignment with the ESC 2023 guidelines on ACS management, which include the CV-polypill (class of recommendation: IIa, level of evidence: B), should commence as early as possible to enhance clinical outcomes, improve patients’ quality of life, and promote adherence (5).

Panelists agreed that hospital discharge from the cardiology ward or immediate follow-up visits are appropriate opportunities to initiate or transition patients to a polypill containing ASA, atorvastatin, and ramipril. Hospital discharge planning and practices hold significant importance for two primary reasons. First, particularly after an AMI, recommendations made at discharge carry considerable weight due to their critical timing, emotional impact, and the comprehensive guidance healthcare providers can offer to patients (20). Second, studies demonstrate that patients discharged from cardiology wards post-AMI exhibit higher treatment adherence levels compared to those discharged from other wards for up to 2 years following the acute event (21). Therefore, effective practices in hospital discharge processes play a pivotal role in influencing adherence to cardioprotective medications for at least 2 years post-discharge (21).

In line with clinical guidelines (4,5,22), panelists stressed the importance of hospital discharge recommendations encompassing lifestyle adjustments, patient-centered education, and support network involvement for successful home management. They unanimously emphasized engaging multidisciplinary cardiology teams proficient in the therapeutic benefits of the CV-polypill, particularly during medication reconciliation, an essential component of discharge transition. Medication reconciliation ensures patients understand their medications, reducing readmission risk, enhancing adherence, and minimizing adverse events (23). Consensus was also reached on standardized algorithms for polypill initiation or switching, guiding physicians in potential incorporation of other cardioprotective medications and selecting the most suitable presentation of the ASA, atorvastatin, ramipril polypill for individual patients (16,24).

Delphi panelists achieved a consensus on various patient profiles suitable for the polypill treatment, going beyond those with prior coronary ischemic events. Strong agreement emerged in favor of recommending the polypill containing ASA, atorvastatin, and ramipril for patients with a history of ischemic stroke and peripheral artery disease, reflecting its potential role in comprehensive cardiovascular risk reduction. This recommendation aligns with real-world evidence (11,14,25), but it should not be extrapolated to patients with a history of hemorrhagic stroke, for whom ASA may be contraindicated (26).

Panelists also acknowledged high satisfaction among physicians and patients with the polypill, highlighting its clinical significance. However, differing factors influence physician and patient decisions, reflecting the complexity of treatment choices and shared decision-making (27). While physicians prioritize effectiveness in risk reduction, patients tend to emphasize regimen simplification and convenience. Such differences in treatment priorities stress the importance of shared decision-making in secondary prevention. Yet, the extent to which these considerations translate into routine clinical practice is strongly influenced by the healthcare context (28,29).

Cost challenges, and reimbursement disparities, along with varying uptake policies, hinder polypill availability worldwide (30,31). In high-income countries, where regulatory pathways, reimbursement mechanisms, and established secondary prevention infrastructures are in place, the implementation of the polypill is shaped largely by clinical evidence, cost-effectiveness evaluations, and physician prescribing habits (30,31,32). Evidence from Europe, for example, has demonstrated the cost-effectiveness of polypill strategies containing aspirin, statins, and renin–angiotensin system inhibitors in patients with established cardiovascular disease, supporting its integration into guidelines and clinical pathways (33,34,35,36). Furthermore, inclusion in the WHO Essential Medicines List (9) and the World Heart Federation Road Map (10) reflects the alignment of the polypill strategy with broader global priorities for secondary prevention, particularly through the simplification of treatment pathways and the embedding of combination therapy into routine discharge and rehabilitation protocols (29).

In low- and middle-income countries, where the CVD burden is disproportionately high and premature mortality more prevalent, the polypill is recognized as a pragmatic strategy to reduce inequities in access to care (14,37,38). However, adoption faces additional barriers, including out-of-pocket expenditure, limited insurance coverage, variable access to essential cardiovascular medicines, and regulatory heterogeneity (39,40,41). Beyond clinical efficacy, its appeal lies in scalability, affordability, and its capacity to address barriers linked to fragmented health systems, uneven distribution channels, and sociocultural factors influencing medication acceptance (28,29,32,33,34,42). The WHO endorsement is expected to facilitate wider procurement and integration into national formularies, guiding adoption, implementation, and scale-up (29). However, real-world uptake still depends on political commitment, supply chain strengthening, and culturally sensitive implementation strategies (37).

The ambivalence observed in this Delphi consensus, reflected in high percentages of neutral responses or evenly distributed scores between the highest and lowest levels of agreement on statements related to these issues, underlines the wide variability of healthcare scenarios across countries and regions. Although these aspects fall outside the scope of the present study, they represent important considerations in the broader context of polypill adoption. Future research should seek to clarify how these contextual variations shape uptake, sustainability, and long-term outcomes in diverse health systems.

This study included several limitations. The use of evidence-based statements on the CV-polypill treatment may introduce bias, potentially influencing panelists. Methodological constraints, such as sample size, participant selection, and feedback quality, challenge generalization. As the Delphi panel was formed through purposeful expert selection rather than geographic or demographic representativeness, certain regions, particularly low- and middle-income countries as well as wealthier areas of Africa, Asia, or the Americas, may have been underrepresented. This reflects the scope of the Delphi consensus, which prioritized clinical expertise over regional representation. In addition, the proportion of cardiologists who already recommend the CV-polypill (74%) may have introduced selection and confirmation biases, potentially overestimating consensus. Therefore, prior experience with the polypill could also have influenced responses. Future analyses stratifying responses by previous polypill use could help assess the impact of these factors on consensus formation. Moreover, the study did not include independent content validation or pre-testing with target users, and no reliability testing or evidence of construct validity was reported. The development and validation of the questionnaire were conducted solely within the authorship group, without engagement from broader stakeholders such as patients, carers, or non-cardiologist healthcare professionals, which may limit the comprehensiveness and generalizability of the findings. Further, the study did not explore system-level implementation factors, such as organizational barriers or policy-level facilitators. These are important next steps for future work on implementation science and health systems integration.

Conducting consultations in English for non-native speakers may impact comprehension, and Likert scales are susceptible to wording and cultural influences, limiting nuanced understanding. Determining sufficient consensus in the Delphi procedure remains subjective and challenging. Despite its limitations, the Delphi method successfully achieved consensus among medical experts on the adoption of the polypill containing ASA, atorvastatin, and ramipril for post-CVD event treatment in real-world settings. This structured approach enabled specialist contributions and provided a comprehensive perspective on the strategy’s effectiveness in preventing recurrence. The use of evidence-based statements and multiple rounds of expert input strengthened the consensus on the clinical value of the polypill in managing patients with established CVD. Importantly, this consensus addresses a critical decision-making gap: the lack of practical, expert-informed guidance on when and for whom to initiate polypill therapy in everyday clinical practice.

## Conclusion

High consensus among Delphi panelists on treatment with a polypill containing ASA, atorvastatin, and ramipril emphasizes its efficacy as a therapy in secondary CVD prevention. This endorsement guides healthcare professionals in optimizing CV outcomes, marking a significant advancement in the prognosis of ASCVD patients. The findings offer a valuable foundation for informing clinical practice and potentially health policy while identifying areas for further research. Importantly, while in high-income countries the focus may be on integration into guideline-based care and cost-effectiveness thresholds, in low- and middle-income countries the emphasis lies on improving affordability, access, and equity.

## Data Accessibility Statement

All data used in this manuscript will be available to anyone who wishes to access the data immediately following publication and indefinitely by contacting the corresponding author (Dr. Daniel Piñeiro at djpineiro@gmail.com). All relevant documents have been included in the supplementary materials.

## Additional File

The additional file for this article can be found as follows:

10.5334/gh.1466.s1 Supplementary files. Tables S1 to S11 and Figures S1 to S5.
