# Supplementary material for: The Polypill (Acetyl Salicylic Acid, Atorvastatin, and Ramipril) Paradigm Shift in Secondary Prevention: Global Expert Delphi Consensus
Source: Glob Heart. 2025 Sep 24;20(1):83. doi: 10.5334/gh.1466 (PMC12466114; doi:10.5334/gh.1466)
Supplement: Supplementary files. — Tables S1 to S11 and Figures S1 to S5. [file gh-20-1-1466-s1.pdf]

Supplementary material

Supplementary tables

Table S1. CREDES checklist

Table S2. ACCORD checklist

Table S3. Search strategy for the systematic review of the literature

Table S4. Selection criteria for the systematic review of the literature

Table S5. Demographic characteristics of Delphi panellists

Table S6. Statements without consensus in round 1, revised and reworded for reassessment in round 2

Table S7. Level of agreement achieved for each statement after Delphi consultation round 2

Table S8. Statements without consensus after round 2

Table S9. Level of agreement with statement 13 per region

Table S10. Panellists' ranking of importance of factors determining patients' adherence to the cardiovascular polypill*

Table S11. Pearson correlation between Delphi participants' agreements on statements to start or switch to the cardiovascular polypill and statements on its efficacy, effectiveness, cost-effectiveness and inclusion in the therapeutic plan

Supplementary figures

Figure S1. PRISMA diagram of the systematic review of the literature

Figure S2. Example of a revised statement assessed in Delphi consultation round 2

Figure S3. Consensus statements flow

Figure S4. Percentage of Delphi panellists agreeing on the use of the cardiovascular polypill* in patients with various clinical profiles

Figure S5. Percentage of Delphi panellists agreeing on the importance of factors related to decisions about using the cardiovascular polypill* in CHD patients

Supplementary tables

Table S1. CREDES checklist (1)

		**Page (line number)**
Transparency and quality of reporting	Purpose well defined	Page 6 (116-119)
	Rationale for Delphi (2–4)	Page 6 (125-128)
	Selection of experts clearly justified	Page 78(170-1178)
	Clear description of methods	Page 6-9
	Flow chart	Figure S3
	Clear definition of consensus (5–7)	Page 8 (160-169)
	Pilot test of instruments	Page 7 (149-155)
	Transparent reporting of results	Page 9-13
	Data analysis clearly justified and reported (8)	Page 8 (196-204)
	Information of rounds (9,10)	Page 6-7 (130-168)
	Discussion of limitations	Page 16-17 (441-462)
	Adequacy of conclusions	Page 17 (484-489)
Selection criteria expert panel	Member of organisation	Page 8 (170-178)
	Recognised authority	Page 8 (170-178)
	Relevant clinical academic expertise	Page 8 (170-178)
	Geographical scope	Page 8 ((170-178)
	Setting/work field	Page 8 (170-178)
	Profession/stakeholder	Page 8 (170-178)(
Definition of consensus		
Purpose and number of Delphi rounds	Identification of issues generation of items	Page 6 (130-136)
	Development of a draft document	Page 7 (149-155)
	Rating/evaluation of statements/document (11–13)	Page 7 (149-155)
	Ranking/selection/prioritisation (2,3)	Page 7 (140-145 ()
	Qualitative responses/comments/feedback	Page 7 (157-160)
	Review/approval of (final) framework	Page 7 (149-150)
	Number of rounds	Page 6-7 (130-168)
Feedback and design of next rounds	Statistical group response	Page 9 (215-217)
	Summary of qualitative comments	Table S6
	Inclusion of items newly generated/added by experts	Table S6
	Modification of items	Table S6
	Selection/reduction of items	Table S6
	Presentation of final document for approval	Page 8 (191-193)

Table S2. ACCORD checklist (14)

Item No.	Section	Checklist Item (*help text*)	Page No.
T1	**Title**	Identify the article as reporting a consensus exercise and state the consensus methods used in the title.<br>*For example, Delphi or nominal group technique.*	1
I1	**Introduction**	Explain why a consensus exercise was chosen over other approaches.	125-128
I2		State the aim of the consensus exercise, including its intended audience and geographical scope (national, regional, global).	116-119
I3		If the consensus exercise is an update of an existing document, state why an update is needed, and provide the citation for the original document.	Not applicable
M1	**Methods**<br>Registration	If the study or study protocol was prospectively registered, state the registration platform and provide a link. If the exercise was not registered, this should be stated.<br>*Recommended to include the date of registration.*	497-498
M2	Selection of SC and/or panellists	Describe the role(s) and areas of expertise or experience of those directing the consensus exercise.<br>*For example, whether the project was led by a chair, co-chairs or a steering committee, and, if so, how they were chosen. List their names if appropriate, and whether there were any subgroups for individual steps in the process.*	149-150
M3		Explain the criteria for panellist inclusion and the rationale for panellist numbers. State who was responsible for panellist selection.	170-182
M4		Describe the recruitment process (how panellists were invited to participate).<br>*Include communication/advertisement method(s) and locations, numbers of invitations sent, and whether there was centralised oversight of invitations or if panellists were asked/allowed to suggest other members of the panel.*	170-178
M5		Describe the role of any members of the public, patients or carers in the different steps of the study.	Not applicable
M6	Preparatory research	Describe how information was obtained prior to generating items or other materials used during the consensus exercise.<br>*This might include a literature review, interviews, surveys, or another process.*	132-136
M7		Describe any systematic literature search in detail, including the search strategy and dates of search or the citation if published already.<br>*Provide the details suggested by the reporting guideline PRISMA and the related PRISMA-Search extension.*	Supp. Table S2 and S3 Figure S1
M8		Describe how any existing scientific evidence was summarised and if this evidence was provided to the panellists.	137-139
M9	Assessing consensus	Describe the methods used and steps taken to gather panellist input and reach consensus (for example, Delphi, RAND-UCLA, nominal group technique).<br>*If modifications were made to the method in its original form, provide a detailed explanation of how the method was adjusted*	157-160

Item No.	Section	Checklist Item (*help text*)	Page No.
		and why this was necessary for the purpose of your consensus-based study.	
M10		Describe how each question or statement was presented and the response options. State whether panellists were able to or required to explain their responses, and whether they could propose new items. *Where possible, present the questionnaire or list of statements as supplementary material.*	140-145<br>162-168
M11		State the objective of each consensus step. *A step could be a consensus meeting, a discussion or interview session, or a Delphi round.*	125-168
M12		State the definition of consensus (for example, number, percentage, or categorical rating, such as 'agree' or 'strongly agree') and explain the rationale for that definition.	184-193
M13		State whether items that met the prespecified definition of consensus were included in any subsequent voting rounds.	162-163
M14		For each step, describe how responses were collected, and whether responses were collected in a group setting or individually.	196-198
M15		Describe how responses were processed and/or synthesised. *Include qualitative analyses of free-text responses (for example, thematic, content or cluster analysis) and/or quantitative analytical methods, if used.*	196-204
M16		Describe any piloting of the study materials and/or survey instruments. *Include how many individuals piloted the study materials, the rationale for the selection of those individuals, any changes made as a result and whether their responses were used in the calculation of the final consensus. If no pilot was conducted, this should be stated.*	149-155
M17		If applicable, describe how feedback was provided to panellists at the end of each consensus step or meeting. *State whether feedback was quantitative (for example, approval rates per topic/item) and/or qualitative (for example, comments, or lists of approved items), and whether it was anonymised.*	162-168
M18		State whether anonymity was planned in the study design. Explain where and to whom it was applied and what methods were used to guarantee anonymity.	178-182
M19		State if the steering committee was involved in the decisions made by the consensus panel. *For example, whether the steering committee or those managing consensus also had voting rights.*	149-150
M20	Participation	Describe any incentives used to encourage responses or participation in the consensus process. *For example, were invitations to participate reiterated, or were participants reimbursed for their time.*	178-182
M21		Describe any adaptations to make the surveys/meetings more accessible. *For example, the languages in which the surveys/meetings were conducted and whether translations or plain language summaries were available.*	158

Item No.	Section	Checklist Item (*help text*)	Page No.
R1	Results	State when the consensus exercise was conducted. List the date of initiation and the time taken to complete each consensus step, analysis, and any extensions or delays in the analysis.	157-162
R2		Explain any deviations from the study protocol, and why these were necessary. *For example, addition of panel members during the exercise, number of consensus steps, stopping criteria; report the step(s) in which this occurred.*	216-217
R3		For each step, report quantitative (number of panellists, response rate) and qualitative (relevant socio-demographics) data to describe the participating panellists.	215-217
R4		Report the final outcome of the consensus process as qualitative (for example, aggregated themes from comments) and/or quantitative (for example, summary statistics, score means, medians and/or ranges) data.	214-348
R5		List any items or topics that were modified or removed during the consensus process. Include why and when in the process they were modified or removed.	Figure S3
D1	Discussion	Discuss the methodological strengths and limitations of the consensus exercise. *Include factors that may have impacted the decisions (for example, response rates, representativeness of the panel, potential for feedback during consensus to bias responses, potential impact of any non-anonymised interactions).*	441-462
D2		Discuss whether the recommendations are consistent with any pre-existing literature and, if not, propose reasons why this process may have arrived at alternative conclusions.	350-439
O1	Other information	List any endorsing organisations involved and their role.	550
O2		State any potential conflicts of interests, including among those directing the consensus study and panellists. Describe how conflicts of interest were managed.	551
O3		State any funding received and the role of the funder. *Specify, for example, any funder involvement in the study concept/design, participation in the steering committee, conducting the consensus process, funding of any medical writing support. This could be disclosed in the methods or in the relevant transparency section of the manuscript. Where a funder did not play a role in the process or influence the decisions reached, this should be specified.*	550

Table S3. Search strategy for the systematic review of the literature (28,30–34)

#	Search terms, strategy	Pubmed, hits (n)	Scopus, hits (n)
	Limit all searches to English, Spanish languages.<br>Limit searches and to dates from 2003 to 2022. (First publication on the polypill concept in 2003 (15))		
**1**	Coronary heart disease		
**2**	Cardiovascular disease		
**3**	Heart disease		
**4**	Secondary prevention		
**5**	Polypill		
**6**	Fixed-dose combination		
**7**	Efficacy		
**8**	Safety		
**9**	Effectiveness		
**10**	Cost-effectiveness		
**11**	Blood pressure		
**12**	Triglycerides		
**13**	Lipids		
**14**	Cholesterol		
**15**	Adherence		
**16**	Compliance		
**17**	Preference		
**18**	Satisfaction		
**19**	Persistence		
**20**	Consensus		
**21**	#2 AND #4 AND #5	133	149
**22**	#4 AND #5		175
**23**	#2 AND #3 AND #5	48	
**24**	#2 AND #4 AND #6	87	
	#2 AND #5 AND #7	67	
**25**	#2 AND #5 AND #8	42	
**26**	#2 AND #5 AND #9	69	
**27**	#2 AND #5 AND #10	55	90
**28**	#4 AND #5 AND #11	47	
**29**	#4 AND #5 AND #12	2	
**30**	#4 AND #5 AND #13	23	
**31**	#4 AND #5 AND #14	42	
**32**	#2 AND #5 AND #15 OR #19	161	153
**33**	#2 AND #5 AND #16		170
**34**	#2 AND #5 AND #17	3	4
**35**	#2 AND #5 AND #18	4	5
**36**	#2 AND #5 AND #20	15	11
	**Total hits per database (all search strategies)**	798	757
	**Total hits**	1555	

Table S4. Selection criteria for the systematic review of the literature

**Inclusion criteria**	**Exclusion criteria**
• Secondary prevention population: representative of general adult population with history of ischaemic cardiovascular disease<br>• Clinical trials (experimental) or observational studies (real world evidence))<br>• Systematic reviews and meta-analysis<br>• Guidelines, position, and consensus documents*<br>• Studies involving a polypill with the components: lipid-lowering drug, antihypertensive and antiplatelet drugs<br>• Polypill efficacy/effectiveness with any of the following end points: cardiovascular death, nonfatal type 1 myocardial infarction, nonfatal ischaemic stroke, coronary revascularisation, adherence/persistence, time to recurrent cardiovascular event, systolic blood pressure, total- / HDL- / LDL-cholesterol<br>• Cost-effectiveness studies<br>• Satisfaction and preference studies<br>• Available in English or Spanish<br>• Worldwide	• Primary prevention of cardiovascular disease**<br>• Study design, rationale and methods only articles<br>• Editorials, comments and opinion letters<br>• Narrative reviews<br>• Studies of polypill components only administered separately<br>• Publications on epidemiology of cardiovascular disease<br>• Cost-effectiveness studies of diagnostic or interventional techniques<br>• Studies evaluating the economic burden of cardiovascular or cerebrovascular diseases<br>• Willingness to pay for health-related quality of life gains CVD studies<br>• Preference-weighted health-related quality of life CVD studies<br>• Quality of life studies in other CVD patient population different from secondary prevention patients<br>• Quality of life studies in very specific CVD patient population (e.g. patients with neurological impairment after stroke)

CVD: Cardiovascular Disease; HDL: High Density Lipoprotein; LDL: Low Density Lipoprotein

*Guidelines, position and consensus documents are considered in this review for the solely reason of identifying aspects on the use of the CV polypill already agreed on in previous publications to avoid repetitions

**Studies covering primary and secondary prevention population will be included considering only the results in secondary prevention

Table S5. Demographic characteristics of Delphi panellists

Characteristic	Value
**Age (SD)**	55.3 years (±9.6)
**Gender, N (%)**	
Female	14 (37%)
Male	24 (63%)
**Continent/country, N (%)**	
**Asia**	**5 (13.2%)**
Armenia	3 (7.9%)
Kazakhstan	2 (5.3%)
**Europe**	**19 (50%)**
Belgium	2 (5.3%)
Germany	1 (2.6%)
Greece	3 (7.9%)
Italy	1 (2.6%)
Portugal	4 (10.5%)
Republic of Belarus	1 (2.6%)
Spain	5 (13.2%)
Ukraine	2 (5.3%)
**Latin America**	**14 (36.8%)**
Chile	3 (7.9%)
Costa Rica	1 (2.6%)
Dominican Republic	1 (2.6%)
Guatemala	1 (2.6%)
Mexico	6 (15.8%)
Honduras	2 (5.3%)
**Medical specialty, N (%)**	
Primary care	0 (0%)
Cardiology	31 (82%)
Neurology	2 (5%)
Internal Medicine	5 (13%)
**Frequency of cardiovascular polypill* recommendation, N (%)**	
Never	0 (0%)
Sometimes	7 (18%)
Frequently	15 (40%)
Very frequently	13 (34%)
Always	3 (8%)

SD: standard deviation

*Cardiovascular polypill developed by the Centro Nacional de Investigaciones Cardiovascualres (CNIC), also known as CNIC-polypill. This polypill contains acetylsalicylic acid (100mg), atorvastatin (20 or 40mg), and ramipril (2.5, 5, or 10mg) and it is commercialized in 23 countries as Trinomia®, Sincronium® or Iltria®

Table S6. Statements without consensus in round 1, revised and reworded for reassessment in round 2

Nº	Original statement/item	Level of agreement in round 1	Participants comments	Arguments from the literature supporting the statement/item	Revised statement/item in round 2
#6	Patients' adherence to CVD secondary prevention treatment two years after starting is lower than 50%.	71%	- *"Not sure if is 50%. Maybe in this objective is 50% but more than 50% is taking lipid lowering therapies and antiplatelet"*<br>- *"That probably depends on the reason for starting secondary prevention as well as other factors like the ones mentioned in statement 5"*<br>- *"Depends on the contact of medical staff and patient, also on patient education"*<br>- *"In my daily clinical practice the number of patients compliant to the treatment is greater than 50%"*<br>- *"The adherence observed in the SECURE trial after 24 months was above 70%"*<br>- *"This statement is based on information of the FOCUS study done with Prof Castellano et al. In this study the drug adherence was estimated by Morisnky Questionnaire but the adherence by pill account was higher. Adherence can be less than 50% at two years in patients undergoing cv secondary "*<br>- *"Poor adherence, even more so in Latin America and even more so in real life"*<br>- *"After 2 years most of patients has lower than 40% of adherence "*	- P Patients with CVD treated with a triple free drug combination were non-adherent in 50.6% after one year follow-up (16)<br>- In the SECURE trial the adherence was 74.1% in the polypill group and 63.2% in the usual care group after 2 years of treatment (17).<br>- Post myocardial infarction patients fully adhering to medication (taking prescribed drugs >80% of days) have lower incidence of major adverse cardiovascular events than those with partial (40-80% of days covered) or no adherence (18).	Patients' adherence to CVD secondary prevention treatment two years after starting is variable amongst individuals and can be lower than 50%.
#9c	Patient satisfaction with the cardiovascular polypill treatment is determined by infrequent adverse events	68.4%	- *"the effectiveness is a concept not well perceived by the patient"*<br>- *"I understand that the convenience is not clearly different than the option a) (Usually patients consider that polypill is more convenient due to the reduced number and frequency of pills) and that is why I have chosen the option Neither agree nor disagree. Regardless high effectiveness, patients usually chose the option recommended by their doctors who really have the information about effectiveness."*	- The effectiveness score encompasses medication ability to prevent or treat the disease, relief of symptoms and the time the medications starts working (17,19).<br>- The effectiveness score is higher in patients with the polypill than monocomoponents (75.02 vs 67.55) after a mean treatment time of 3 years (19)<br>- The effectiveness score in the treatment satisfaction questionnaire in the SECURE trial was higher after 24 months of treatment	In secondary prevention patients, satisfaction with the cardiovascular polypill strategy relates to its ability to prevent events, relieve symptoms and the time it takes to start working.

Nº	Original statement/item	Level of agreement in round 1	Participants comments	Arguments from the literature supporting the statement/item	Revised statement/item in round 2
			- *"Although high effectiveness is a critical parameter for clinicians, patients are not always sufficiently aware of the meaning of this parameter. Even when they mention it as an important parameter they often do not know what it really represents in clinical terms. Together with infrequent adverse events these are for sure important determinants but unfortunately, in clinical practice, for many patients, not the primary ones when compared to the cost of the medication, for example. "*	(mean difference: 5.26, 95%CI: 3.59,6.94) (17)	
#9d	Patient satisfaction with the cardiovascular polypill treatment is determined by infrequent adverse events	63.2%	- *"Patient satisfaction in terms of perceived efficacy and risk of adverse events is similar to satisfaction with standard therapy. "*<br>- *"I think that number of adverse events is the same"*<br>- *"More Side effects will be expected in patients with higher adherence, as shown in SECURE trial. "*<br>- *"The difference between the two groups even having a difference of 5% favouring the polypill I don't think that the difference will be assessed by side effects because there were not statistical difference between these groups"*<br>- *"We did not find the proof of this statement : fewer adverse events in attached references. Adverse events were comparable in both arm medication strategies"*	- The global satisfaction domain encompasses medication is good for the patient, medication outweigh the bad things and satisfaction with the medication (17,19)<br>- The global satisfaction score was significantly higher in the polypill cohort versus monocomponents (77.3 vs 71.2) after 3 years of treatment (19)<br>- The global satisfaction score in the SECURE trial is higher than with monocomponents (mean difference: 6.60; 95%CI: 4.93,8.27) (17)	In secondary prevention patients, global satisfaction with the cardiovascular polypill relates to the perception that the clinical benefits outweigh the potential side effects.
#13	Many patients (around 60%) will switch to the polypill even if it is the more expensive option.	50%	- *"Cost always is important for the patient and one registry could not be the main study to conclude this"*<br>- *"That will depend on the economic situation of the patient"*<br>- *"Polypill costs may impact patients switch to the polypill, especially if the polypill is not reimbursed by health care system"*<br>- *"The patients will look for cheap options"*	- Cost is one of the most common reasons for non-adherence (20)	The acquisition costs of not reimbursed medications can be a barrier to patients. Assuming financial capacity, more than a half of secondary CVD prevention patients would switch to the polypill even if it is a more expensive option.

Nº	Original statement/item	Level of agreement in round 1	Participants comments	Arguments from the literature supporting the statement/item	Revised statement/item in round 2
			- *"In real life situations, cost would definitely are going to be a barrier"*<br>- *"The same comment of statement 12. This study incorporated few patients an have many limitations. The information is interesting but cannot be consider as a strong evidence. "*<br>- *"Need for more robust evidence in clinical practice to prove that these high rates are reproducible"*<br>- *"In my opinion, there are not enough evidence to support this statement. We neeed more studies, especially in low-income countries, to affirm that polypill would be the preferred option even it was the most expensive one. "*<br>- *"63.10%"*		
#15a and 15b	Compared with monocomponents given separately, patients undergoing the polypill treatment experience smaller number of visits to the general practitioner and to the specialist	57.9%	- *"I think that this points are correct for both strategies".*<br>- *"It depends on status of the disease and comorbidities"*<br>- *"The number of visits in the polypill does not necessarily represents less number of CV events "*<br>- *"This is specially related to public health and in not applicable to private practice"*<br>- *"Once again, the pharmacological synergy in a polypill has benefits over the use of individual medication, and this, the medication with individual and non-synergistic tablets, is the most used strategy in public policies in Latin American countries, which is why it is a good point of improvement. and a good action strategy that will benefit everyone"*<br>- *"Although some studies have shown that polypill strategy, in comparison with monocomponents therapies, can reduce the number of visits both to PC and specialists, I believe that we need more studies in different settings and different health care systems"*	- Several studies confirm the reduction of visits to the general practitioner:<br>- Visits to primary care in the polypill were 16.6 versus 18.7 (monocomponents), 18.9 (equipotent medications) and 21.0 (usual care); p<0.001 (22)<br>- Visits to the specialist in the polypill were 5.0 versus 6.2 (monocomponents), 6.5 (equipotent medications) and 7.3 (usual care); p<0.001 (22)<br>- The cost of patient management is 7.4% less in the polypill group compared to monocomponents (23)<br>- The use of the polypill reduces the direct incremental cost corresponding to patient management compared to monocomoponents (24,25)	Compared with monocomponents given separately, the costs cardiovascular disease management and follow up decrease amongst secondary prevention patients undergoing the polypill treatment.

Nº	Original statement/item	Level of agreement in round 1	Participants comments	Arguments from the literature supporting the statement/item	Revised statement/item in round 2
			- *"I will be cautious with the result of the study of Wilke et al, in the regard of the reduction to GP visits. Even though the p value is 0.03 the confidence intervals were 0.84-1.01, and the rate ratio was 0.91 the lowest and less significant result"*<br>- *"Again, patients receiving polypill may have many different pathologies for which they may need to be visited by specialists, and none to them will be reduced by taking polypill"*<br>- *"According to the Wilke et al. study results* (21), *all-cause specialist visits rate ratios associated with polypill was between 0.645 and 0.962; 95% CIs: between 0.50 and 1.08; p-values: between <0.001 and 0.073), with the exception of patients receiving EZE/ATOR (rate ratio: 1.128; 95% CI: 0.92–1.38; p > 0.10. These results, which are obtained with an observational study, are not strong enough to support Statement 15, b"*		
#15c	Compared with monocomponents given separately, patients undergoing the polypill treatment experience shorter all-cause hospitalisations (in days)	67.6%	- *"More evidence is needed"*	- Several studies confirm the reduction of days of hospitalization:<br>- Days of hospitalization in the polypill were 2.3 versus 3.4 (monocomponents), 3.7 (equipotent medications) and 4.0 (usual care); p<0.001 (22).<br>- The use of the polypill reduces the cost of acute cardiovascular events and the cost of patient's management compared to monocomoponents (23).<br>-	Compared with monocomponents given separately, the use of healthcare resources such as all-cause hospitalizations is smaller amongst patients undergoing the polypill treatment

Nº	Original statement/item	Level of agreement in round 1	Participants comments	Arguments from the literature supporting the statement/item	Revised statement/item in round 2
#16	Compared to monocomponents given separately, the cardiovascular polypill is more effective at preventing:<br>a. Recurrent CHD events in women and men alike<br>b. Recurrent strokes in women and men alike	78.9%	- *"I cannot see may scientific reasons why polypill should be more effective in women. "*<br>- *"The reference is of a cost-effective analysis, don´t understand how it can be extrapolated to a clinical statement. Moreover, it concerns a cost-effectiveness model applied only to Portugal. "*<br>- *"I read the publication of Aguiar et al (Cost-Effectiveness of the CNIC-Polypill Strategy Compared With Separate Monocomponents in Secondary Prevention of Cardiovascular and Cerebrovascular Disease in Portugal: The MERCURY Study). In this publication, the authors concluded that the CNIC polypill is more cost effective to control CVD risk factors and reduce events (CHD and cerebrovascular) lifelong. Nevertheless in this study there is no sex analysis. Around 60 % of the study population were men., and the authors said that they used the SMART risk equation, which is based on age, gender, and clinical parameters in a Cox proportional hazards model to calculate the occurrence of major CV events (MI, stroke, or CV death). I am a clinician and not expert on cost-effectiveness analysis, so perhaps this model can confirmed that the result is the same for women and men. From my clinical point of view, I think that we must be cautious with that sentence. I read the publication of Aguiar et al (Cost-Effectiveness of the CNIC-Polypill Strategy Compared With Separate Monocomponents in Secondary Prevention of Cardiovascular and Cerebrovascular Disease in Portugal: The MERCURY Study). In this publication, the authors concluded that the CNIC polypill is more cost effective to control CVD risk factors and reduce events (CHD and cerebrovascular) lifelong. Nevertheless in this study there is no sex analysis. Around 60 % of the study population were men., and the authors said*	- Higher reduction of SBP was achieved with the polypill than with usual care. The polypill prevents recurrent stroke after hospital discharge, due to better lowering of BP (26)<br>- In patients with either a previous CHD event or a stroke, the CNIC-polypill prevents subsequent CHD events, recurrent strokes, and CV deaths vs monocomponents over the patient's lifetime (25).<br>- The proportion of patients with controlled blood pressure after 2 years of therapy was higher with the CNIC-Polypill compared to each of the other cohorts [SBP/DBP <130/80mmHg: 44.1% vs 37.9 % (p<0.05); 34.6% (p< 0.01) and 32.4% (p<0.01) monocomponents, equipotents and other therapies, respectively (27).<br>- Mean time to recurrent MACE is longer with the CNIC-Polypill compared to control (27)<br>- Polypill, with or without aspirin, improves risk factors control and cardiovascular outcomes in primary prevention, reducing cardiovascular mortality and major cardiovascular events in comparison with placebo or no pharmacologic treatment (28).	Compared to monocomponents given separately, the cardiovascular polypill is more effective at preventing recurrent strokes mainly due to better SBP control irrespective of gender

Nº	Original statement/item	Level of agreement in round 1	Participants comments	Arguments from the literature supporting the statement/item	Revised statement/item in round 2
			that they used the SMART risk equation, which is based on age, gender, and clinical parameters in a Cox proportional hazards model to calculate the occurrence of major CV events (MI, stroke, or CV death). I am a clinician and not expert on cost-effectiveness analysis, so perhaps this model can confirmed that the result is the same for women and men. From my clinical point of view, I think that we must be cautious with that sentence. To stated that CNIC polypill reduce events in women and men alike is uncertain, because the impact in decreasing CHD and cerebrovascular disease of different drugs is not exactly on both sexes. In fact, in the SECURE study the primary composite outcome according to sex showed that in men polypill strategy was better, instead in women showed a trend. ”<br>- *“These statements are not reflected in cited study of Aguiar”*<br>- *“O not know if they are alike in women and men, and i did not find it”*		
#22	The cardiovascular polypill treatment should be implemented on hospital discharge, per protocol, as a core therapy option for all CHD secondary prevention patients with no contraindications.	78.9%	- *”On hospital discharge there could be some difficulties to prescribe polypill, it can be done during the first follow-up visit. ”*<br>- *”Not per protocol”*<br>- *”An small group of patients need specific treatment and adjust dose to achieve goals”*<br>- *”even strongly agree. we’ve executed some practical single centre investigation upon the topic through 2018-2022. Near 24% on discharge after ACS have clear indications”*<br>- *”I agree with the protocol suggested by Grigorian et al. It looks very reasonable, and the contraindications are the most frequent*	- The suitability of the cardiovascular polypill at discharge/rehabilitation depends on the patient’s profile (29)<br>- According to ESC guidelines, the monocomponents and antiplatelet therapy (P2Y12 inhibitor) are the routine pharamacological therapy (30)<br>- A cardiovascular polypill is a valid option after an atherosclerotic cardiovascular event, arising from coronary, cerebrovascular, or peripheral arterial disease, in patients with indication for the composite of the monocomponents, assuming that this strategy can improve treatment adherence (28)	The cardiovascular polypill could be implemented on hospital discharge or first follow up visits as the core therapy option for all CHD secondary prevention patients with no contraindications.

Nº	Original statement/item	Level of agreement in round 1	Participants comments	Arguments from the literature supporting the statement/item	Revised statement/item in round 2
			presented as the use of anticoagulants or the need of ENTRESTO, and obviously the intolerance to one of components of the polypill"<br>- *"Not should, it could switch the word should for could"*		

Table S7. Level of agreement achieved for each statement after Delphi consultation round 2 (from highest to lowest)

**Objectives and recommendations for the cardiovascular polypill* on everyday practice and hospital discharge**						
**Nº**	**Reference**	**Statement**	**Agree % (n)¥**	**Neither agree nor disagree % (n) ¥**	**Disagree % (n) ¥**	**Valid answers**
#22	Grigorian 2021 (29)	The cardiovascular polypill* could be implemented on hospital discharge or first follow up visits as a baseline therapy option for all CHD secondary prevention patients with no contraindications.	100.0 (37)	0.0 (0)	0.0 (0)	37
#25	Expert opinion	All members of a hospital multidisciplinary cardiology team should be aware of the benefits of the cardiovascular polypill* in the secondary prevention of CVD.	100.0 (38)	0.0 (0)	0.0 (0)	38
#26	Grigorian 2021 (29)	On hospital discharge, the cardiovascular polypill* should be accompanied by the below recommendations to reduce the CVD risk factors and avoid major acute cardiovascular events.				
		a. Healthy diet	100.0 (38)	0.0 (0)	0.0 (0)	38
		b. Smoking cessation	100.0 (38)	0.0 (0)	0.0 (0)	38
		c. Daily physical activity	97.4 (37)	2.6 (1)	0.0 (0)	38
#1	Castellano 2022 (17)	Compared to usual care, the prescription of the cardiovascular polypill* reduces, by 24% over 3 years, the relative risk of cardiovascular death, acute myocardial infarction, stroke, or urgent revascularisation in patients undergoing CHD secondary prevention treatment.	97.4 (37)	2.6 (1)	0.0 (0)	38
#4	Castellano 2022 (17)	The cardiovascular polypill* and usual care are equally safe (the same reported adverse events, magnitude and frequency) for the CVD secondary prevention patients.	97.4 (37)	2.6 (1)	0.0 (0)	38
#28	Adapted from Coca 2020 (31)	On hospital discharge, the following steps should be followed to switch to the cardiovascular polypill* y from monocomponents treatment:<br>1. Revise the current medication<br>2. Decide to switch from monocomponents to the cardiovascular polypill*<br>3. Adapt the doses of antihypertensives and statins to achieve the recommended blood pressure and the target LDL-cholesterol values<br>4. If the patient is treated with an angiotensin receptor blocker, search for an equivalent effective daily dose of ramipril [referring to tables published in Coca 2020 (16)]<br>5. If the patient is treated with a statin different from atorvastatin, search for the equivalent effective daily dose of atorvastatin [referring to tables published in Coca 2020 (16)]<br>6. Adjust the dose of statins if needed and well tolerated<br>7. Add other antihypertensive, ezetimibe or proprotein convertase subtilisin/kexin-type inhibitors, if needed.<br>8. Add the specific drugs for comorbidities<br>9. Check for contraindications/interactions	97.4 (37)	2.6 (1)	0.0 (0)	38

Nº	Reference	Statement	Agree % (n)[¥]	Neither agree nor disagree % (n) [¥]	Disagree % (n) [¥]	Valid answers
#23	Grigorian 2021 (29)	The cardiovascular polypill* should be a core therapy for secondary prevention patients on hospital discharge to:				
		a. Increase adherence	94.7 (36)	5.3 (2)	0.0 (0)	38
		b. Reach treatment objectives effectively and safely	81.6 (31)	18.4 (7)	0.0 (0)	38
#30	Adapted from AHRQ 2022 (32)	The cardiovascular polypill* discharge planning should:				
		Listen to the patient's and family's goals, preferences, observations and concerns about adopting the treatment with the cardiovascular polypill*	97.4 (37)	2.6 (1)	0.0 (0)	38
		Educate the patient and family, using plain language, about the patient's condition, the discharge process, the cardiovascular polypill* concept and the value of the polypill strategy in terms of efficacy, effectiveness, safety and treatment simplification	94.7 (36)	5.3 (2)	0.0 (0)	38
		Discuss with the patient and family five key areas to prevent problems with the treatment with the cardiovascular polypill* at home:<br>1. Describe their everyday life, including the secondary prevention treatment at home<br>2. Review medications: explain the objectives of the cardiovascular polypill*, the concomitant medication, and the importance of persistence and good adherence to the cardiovascular polypill* to prevent recurrent events<br>3. Highlight warning signs and possible problems: explain interactions and potential side-effects<br>4. Explain test results, including LDL-cholesterol, triglycerides, apolipoproteins and sugar levels<br>Make follow-up appointments to revise the treatment plan and the use of the cardiovascular polypill* at home	92.1 (35)	7.9 (3)	0.0 (0)	38
		Include the patient and family as full partners in the discharge planning process, incorporating the cardiovascular polypill* in the transition from hospital to home	91.9 (34)	8.1 (3)	0.0 (0)	37
		Assess how well the doctors and nurses explain the diagnosis, the condition and the treatment with the cardiovascular polypill* to prevent recurrent events and use the teach-back method	89.5 (34)	10.5 (4)	0.0 (0)	38
#16	Aguiar 2022 (25)	Compared to monocomponents given separately, the cardiovascular polypill* is more effective at preventing:				
		a. Recurrent strokes mainly due to better SBP control irrespective of gender	89.2 (33)	8.1 (3)	2.7 (1)	37
		b. Recurrent CHD events in women and men alike	84.2 (32)	15.8 (6)	0.0 (0)	38
#24	Castellano 2022 (17)	In a patient with an acute coronary event, the cardiovascular polypill* should be started within 8 days of the event or as soon as possible after stabilisation.	81.1 (30)	16.2 (6)	2.7 (1)	37

Cost effectiveness of the cardiovascular polypill* strategy						
**Nº**	**Reference**	**Statement**	**Agree % (n)¥**	**Neither agree nor disagree % (n) ¥**	**Disagree % (n) ¥**	**Valid answers**
#15	Wilke 2022 (21)	Compared with monocomponents given separately, patients undergoing the polypill treatment experience a decrease in the costs of cardiovascular disease and follow up	91.9 (34)	8.1 (3)	0.0 (0)	37
#17	Aguiar 2022 (25)	Compared to monocomponents given separately, the cardiovascular polypill* prevents recurrent CHD events and strokes at an affordable cost to the healthcare system.	89.5 (34)	2.6 (1)	7.9 (3)	38
#14	Marquina 2022 (33)	From a societal perspective, the polypill strategy delivers significant cost savings over the patient's lifetime, compared to monocomponents given separately, due to:				
		a. Reduction in CVD events	89.5 (34)	10.5 (4)	0.0 (0)	38
		b. Fewer hospitalisations	89.5 (34)	7.9 (3)	2.6 (1)	38
		c. Fewer productivity loss	89.5 (34)	5.25 (2)	5.25 (2)	38
#15	Wilke 2022 (21)	Compared with monocomponents given separately, patients undergoing the polypill treatment experience:				
		a. A smaller number of CVD-related prescriptions	89.5 (34)	7.9 (3)	2.6 (1)	38
		b. A smaller use of healthcare resources such as all-cause hospitalizations	80.6 (29)	16.7 (6)	2.8 (1)	36
#18	Jahangiri 2022 (34)	c. Depending on drug prices and reimbursement policies in different countries, improvements in CHD patient adherence to the polypill treatment are achieved at a cost equal to or lower than separately administered multiple monocomponents.	84.2 (32)	13.2 (5)	2.6 (1)	38
#13	Cosin-Sales 2021 (19)	The acquisition costs of not reimbursed medications can be a barrier to patients. Assuming financial capacity, more than a half of secondary CVD prevention patients would switch to the polypill even if it is a more expensive option	70.3 (26)	13.5 (5)	16.2 (6)	37

Patient profile and treatment decision making on the cardiovascular polypill treatment						
**Nº**	**Reference**	**Statement**	**Agree % (n)¥**	**Neither agree nor disagree % (n) ¥**	**Disagree % (n) ¥**	**Valid answers**
#20	Expert opinion	Besides the CHD secondary prevention patients, the cardiovascular polypill* can be effectively and safely recommended for patients with the following profiles:				
		a. Previous stroke patients	94.7 (36)	5.3 (2)	0.0 (0)	38
		b. Peripheral artery disease patients	92.1 (35)	5.3 (2)	2.6 (1)	38
		c. Very high CVD risk patients with no previous acute events	86.8 (33)	10.5 (4)	2.6 (1)	38
		d. High CVD risk patients with no previous acute events	81.6 (31)	15.8 (6)	2.6 (1)	38
		e. Type-2 diabetes mellitus	81.6 (31)	15.8 (6)	2.6 (1)	38
#3	González-Juanatey 2022 (27)	The polypill helps achieve the targets recommended by European guidelines for blood pressure values (< 130/80mmHg) and for LDL-cholesterol (≥ 50%) reduction after 2 years of treatment (from baseline) in patients with established atherosclerotic CVD.	89.5 (34)	5.25 (2)	5.25 (2)	38

Nº	Reference	Statement	Agree % (n)¥	Neither agree nor disagree % (n) ¥	Disagree % (n) ¥	Valid answers
#2	González-Juanatey 2022 (27)	Compared to the monocomponents given separately, 10% to 12% more CHD secondary prevention patients achieve adequate control of blood pressure and LDL-cholesterol levels over 2 years of the cardiovascular polypill* treatment	86.8 (33)	13.2 (5)	0.0.0 (0)	38
**Satisfaction with the cardiovascular polypill***						
**Nº**	**Reference**	**Statement**	**Agree % (n)¥**	**Neither agree nor disagree % (n) ¥**	**Disagree % (n) ¥**	**Valid answers**
#9	Cosin-Sales 2021 (19)	Patient satisfaction with the cardiovascular polypill is determined by the following:				
		a. Simplified treatment regimen (reduced number and frequency of pills)	100.0 (38)	0.0 (0)	0.0 (0)	38
		b. Convenience	94.7 (36)	5.3 (2)	0.0 (0)	38
		c. the perception that the clinical benefits outweigh the potential side effects	86.5 (32)	8.1 (3)	5.4 (2)	37
		d. the ability to prevent events, relieve symptoms and the time it takes to start working.	78.4 (29)	21.6 (8)	0.0 (0)	37
#10	Virdee 2013(35), Murphy 2022 (36)	Physician satisfaction with the cardiovascular polypill is mostly determined by the following:				
		a. Improvement of patient adherence to treatment	100.0 (38)	0.0 (0)	0.0 (0)	38
		b. Remarkable cardiovascular risk reduction	89.7 (34)	7.9 (3)	2.6 (1)	38
		c. Effective achievement of blood pressure and LDL-cholesterol level goals	86.8 (33)	7.9 (3)	5.3 (2)	38
		d. Infrequent and minor adverse events	84.2 (32)	10.5 (4)	5.3 (2)	38
		e. Significant reduction of MACE incidence	84.2 (32)	13.2 (5)	2.6 (1)	38
		f. Robust scientific evidence for CVD secondary prevention	84.2 (32)	10.5 (4)	5.3 (2)	38
		g. Good understanding of the polypill treatment by the patient	81.6 (31)	10.5 (4)	7.9 (3)	38
#11	Cosin-Sales 2021 (19)	Compared with monocomponents given separately, general satisfaction with the cardiovascular polypill* significantly increases after 1 year of treatment amongst CHD secondary prevention patients.	97.4 (37)	0.0 (0)	2.6 (1)	38
#12	Cosin-Sales 2021 (19)	A large percentage of patients (around 70%) will switch to the polypill if offered.	92.1 (35)	7.9 (3)	0.0 (0)	38
	**Patient adherence to the cardiovascular polypill***					
**Nº**	**Reference**	**Statement**	**Agree % (n)¥**	**Neither agree nor disagree % (n) ¥**	**Disagree % (n) ¥**	**Valid answers**
#7	Castellano 2022 (17), González-Juanatey 2022 (27), Cosin-Sales 2021 (19), Castellano 2014 (37)	After 2 years, adherence amongst the patients undergoing cardiovascular polypill* treatment is 10% to 17% higher than amongst those treated with the same monocomponents given separately.	97.4 (37)	2.6 (1)	0.0 (0)	38
#8	Cosin-Sales 2021 (19)	More than 80% of CHD secondary prevention patients find taking the cardiovascular polypill* "very convenient".	94.7 (36)	5.3 (2)	0.0 (0)	38
#6	Castellano 2014 (37)	Patient's adherence two years after starting secondary prevention treatment is variable amongst individuals and can be lower than 50%.	91.9 (34)	2.7 (1)	5.4 (2)	37

¥Calculation based on valid answers
* Cardiovascular polypill developed by the Centro Nacional de Investigaciones Cardiovascualres (CNIC), also known as CNIC-polypill. This polypill contains acetylsalicylic acid (100mg), atorvastatin (20 or 40mg), and ramipril (2.5, 5, or 10mg) and is available in 23 countries as Trinomia®, Sincronium® or Iltria®.
AHRQ: Agency for healthcare Research and Quality, CHD: coronary heart disease, CVD: cardiovascular disease, LDL: Low-density lipoprotein, MACE: major adverse cardiovascular event, SBP: systolic blood pressure

Table S8. Statements without consensus after round 2

Nº	Reference	Statement	Agree % (n) ¥	Neither agree nor disagree % (n) ¥	Disagree % (n) ¥	Valid answers
#13	Cosin-Sales 2021 (19)	The acquisition costs of not reimbursed medications can be a barrier to patients. Assuming financial capacity, more than a half of secondary CVD prevention patients would switch to the polypill even if it is a more expensive option	70.3 (26)	13.5 (5)	16.2 (6)	37
#9c	Cosin-Sales 2021 (19)	Patient satisfaction with the cardiovascular polypill is determined by the ability to prevent events, relieve symptoms and the time it takes to start working.	78.4 (29)	21.6 (8)	0.0 (0)	37

¥Calculation based on valid answers
Cardiovascular polypill developed by the Centro Nacional de Investigaciones Cardiovascualres (CNIC), also known as CNIC-polypill. This polypill contains acetylsalicylic acid (100mg), atorvastatin (20 or 40mg), and ramipril (2.5, 5, or 10mg) and is available in 23 countries as Trinomia®, Sincronium® or Iltria®

Table S9. Level of agreement with statement 13 by region

Region	N	Agree % (n)	Neither agree nor disagree % (n)	Disagree % (n)
Asia	5	60.0% (3)	20.0% (1)	20.0% (1)
Europe	17	64.7% (11)	23.5% (4)	11.8% (2)
Latin America	14	85.7% (12)	0.0% (0)	14.3% (2)
Statement 13: The acquisition costs of not reimbursed medications can be a barrier to patients. Assuming financial capacity, more than a half of secondary CVD prevention patients would switch to the polypill even if it is a more expensive option)				

Table S10. Panellists' ranking of importance of factors determining patients' adherence to the cardiovascular polypill*

Nº	Statement	Rank 1 (Most important, 1 and 2 score) ¥	Rank 2 (Neutral, 3 score)¥	Rank 3 (Least important, 4 and 5 score) ¥
#5	In your view, how important are the following factors for adherence to the cardiovascular polypill* by CVD secondary prevention patients? Please enter a number to indicate the importance of each factor for adherence to the treatment on a scale from 1 to 5. 1 = the most important and 5 = the least important factor			
	Number of tablets taken per day	80.0%	2.9%	17.1%
	Explanation of polypill treatment by the medical staff	71.4%	11.4%	17.1%
	Understanding of polypill treatment by the patient	68.6%	11.4%	20.0%
	Patient's socioeconomic status	68.6%	14.3%	17.1%
	Treatment cost	65.7%	11.4%	22.9%
	Effective communication between doctor and patient	65.7%	14.3%	20.0%
	Presence of comorbidities	62.8%	22.9%	14.3%
	Patient's health literacy	51.4%	34.3%	13.2%
	Patient's age	45.7%	22.9%	31.4%

¥Calculation based on 35 valid answers

*Cardiovascular polypill developed by the Centro Nacional de Investigaciones Cardiovascualres (CNIC), also known as CNIC-polypill. This polypill contains acetylsalicylic acid (100mg), atorvastatin (20 or 40mg), and ramipril (2.5, 5, or 10mg) and is available in 23 countries as Trinomia®, Sincronium® or Iltria®

CVD: cardiovascular disease

Note: Neutral responses are shown for transparency. The "neutral" column reflects participants who rated the statement as moderately important (score 3 in a 1 to 5 scale). A 0.0% indicates clear agreement (e.g., "to reduce risk of MI or stroke"); a higher percentage suggests varying stakeholder views and greater ambivalence about the importance of that statement (e.g., "to reduce treatment cost"). Consider all three columns together to assess the level of agreement

Table S11. Pearson correlation between Delphi participants' agreements on statements to start or switch to the cardiovascular polypill* and statements on its efficacy, effectiveness, cost-effectiveness and inclusion in the therapeutic plan

Nº	Statement	Algorithm TO START the CV-polypill strategy		Algorithm TO SWITCH to the CV-polypill strategy	
		Pearson correlation	p-value	Pearson correlation	p-value
#1	Compared to usual care, the prescription of the cardiovascular polypill* reduces, by 24% over 3 years, the relative risk of cardiovascular death, acute myocardial infarction, stroke, or urgent revascularisation in patients undergoing CHD secondary prevention treatment.	1.00	< 0.001	1.00	< 0.001
#2	Compared to the monocomponents given separately, 10% to 12% more CHD secondary prevention patients achieve adequate control of blood pressure and LDL-cholesterol levels over 2 years of the cardiovascular polypill* treatment.	0.42	0.009	0.42	0.008
#3	The polypill therapy helps achieve the targets recommended by European guidelines for blood pressure values (< 130/80mmHg) and for LDL-cholesterol (≥ 50%) reduction after 2 years of treatment (from baseline) in patients with established atherosclerotic CVD.	0.62	< 0.001	0.62	< 0.001
#7	After 2 years, adherence amongst the patients undergoing cardiovascular polypill* treatment is 10% to 17% higher than amongst those treated with the same monocomponents given separately.	1.00	< 0.001	1.00	< 0.001
#8	More than 80% of CHD secondary prevention patients find taking the cardiovascular polypill* very convenient".	0.69	< 0.001	0.69	< 0.001
#10a	Physician satisfaction with the cardiovascular polypill is mostly determined by significant reduction of MACE incidence	0.66	< 0.001	0.66	< 0.001
#10b	Physician satisfaction with the cardiovascular polypill is mostly determined by effective achievement of blood pressure and LDL-cholesterol level goals	0.59	< 0.001	0.59	< 0.001
#10c	Physician satisfaction with the cardiovascular polypill is mostly determined by remarkable cardiovascular risk reduction	0.75	< 0.001	0.75	< 0.001
#10d	Physician satisfaction with the cardiovascular polypill is mostly determined by robust scientific evidence for CVD secondary prevention	0.56	< 0.001	0.56	< 0.001
#11	Compared with monocomponents given separately, general satisfaction with the cardiovascular polypill* significantly increases after 1 year of treatment amongst CHD secondary prevention patients.	1.00	< 0.001	1.00	< 0.001
#12	A large percentage of patients (around 70%) will switch to the polypill if offered.	0.56	< 0.001	0.56	< 0.001
#17	Compared to monocomponents given separately, the cardiovascular polypill* prevents recurrent CHD events and strokes at an affordable cost to the healthcare system.	0.53	0.001	0.53	< 0.001
#18	Depending on drug prices and reimbursement policies in different countries, improvements in CHD patient adherence to the polypill treatment are achieved at a cost equal to or lower than separately administered multiple monocomponents.	0.66	< 0.001	0.66	< 0.001
#23a	The cardiovascular polypill* should be a core therapy for secondary prevention patients on hospital discharge to reach treatment objectives effectively and safety	0.37	0.021	0.34	0.033
#23b	The cardiovascular polypill* should be a core therapy for secondary prevention patients on hospital discharge to increase adherence	0.67	< 0.001	0.69	< 0.001
#30	The cardiovascular polypill* discharge planning should include the patient and family as full partners in the	0.56	< 0.001	0.56	< 0.001

Nº	Statement	Algorithm TO START the CV-polypill strategy		Algorithm TO SWITCH to the CV-polypill strategy	
		Pearson correlation	p-value	Pearson correlation	p-value
	discharge planning process, incorporating the cardiovascular polypill* in the transition from hospital to home				
#30	The cardiovascular polypill* discharge planning should discuss with the patient and family five key areas to prevent problems with the cardiovascular polypill* at home:<br>1. Describe their everyday life, including the secondary prevention treatment at home<br>2. Review medications: explain the objectives of the cardiovascular polypill*treatment, the concomitant medication, and the importance of persistence and good adherence to the cardiovascular polypill* to prevent recurrent events<br>3. Highlight warning signs and possible problems: explain interactions and potential side-effects<br>4. Explain test results, including LDL-cholesterol, triglycerides, apolipoproteins and sugar levels<br>5. Make follow-up appointments to revise the treatment plan and the use of the cardiovascular polypill* at home	0.69	< 0.001	0.56	< 0.001
#30	The cardiovascular polypill* discharge planning should educate the patient and family, using plain language, about the patient's condition, the discharge process, the cardiovascular polypill* concept and the value of the polypill treatment in terms of efficacy, effectiveness, safety, and treatment simplification	0.69	< 0.001	0.69	< 0.001
#30	The cardiovascular polypill* discharge planning should assess how well the doctors and nurses explain the diagnosis, the condition and the cardiovascular polypill* to prevent recurrent events and use the teach-back method	0.47	0.003	0.47	0.002
#30	The cardiovascular polypill* discharge planning should listen to the patient's and family's goals, preferences, observations, and concerns about adopting the cardiovascular polypill* treatment	1.00	< 0.001	1.00	< 0.001

* Cardiovascular polypill developed by the Centro Nacional de Investigaciones Cardiovascualres (CNIC), also known as CNIC-polypill. This polypill contains acetylsalicylic acid (100mg), atorvastatin (20 or 40mg), and ramipril (2.5, 5, or 10mg) and it is available in 23 countries as Trinomia®, Sincronium® or Iltria®.

CHD: coronary heart disease, CVD: cardiovascular disease, LDL: low-density lipoprotein, MACE: major adverse cardiovascular events

The Pearson correlation coefficient, r, can take a range of values from +1 to -1. A value of 0 indicates that there is no association between the two variables. A value greater than 0 indicates a positive association; that is, as the value of one variable increases, so does the value of the other variable. A value less than 0 indicates a negative association; that is, as the value of one variable increases, the value of the other variable decreases. The stronger the association of the two variables, the closer the Pearson correlation coefficient, r, will be to either +1 or -1 depending on whether the relationship is positive or negative, respectively.

Supplementary figures

Figure S1. PRISMA diagram of the systematic review of the literature

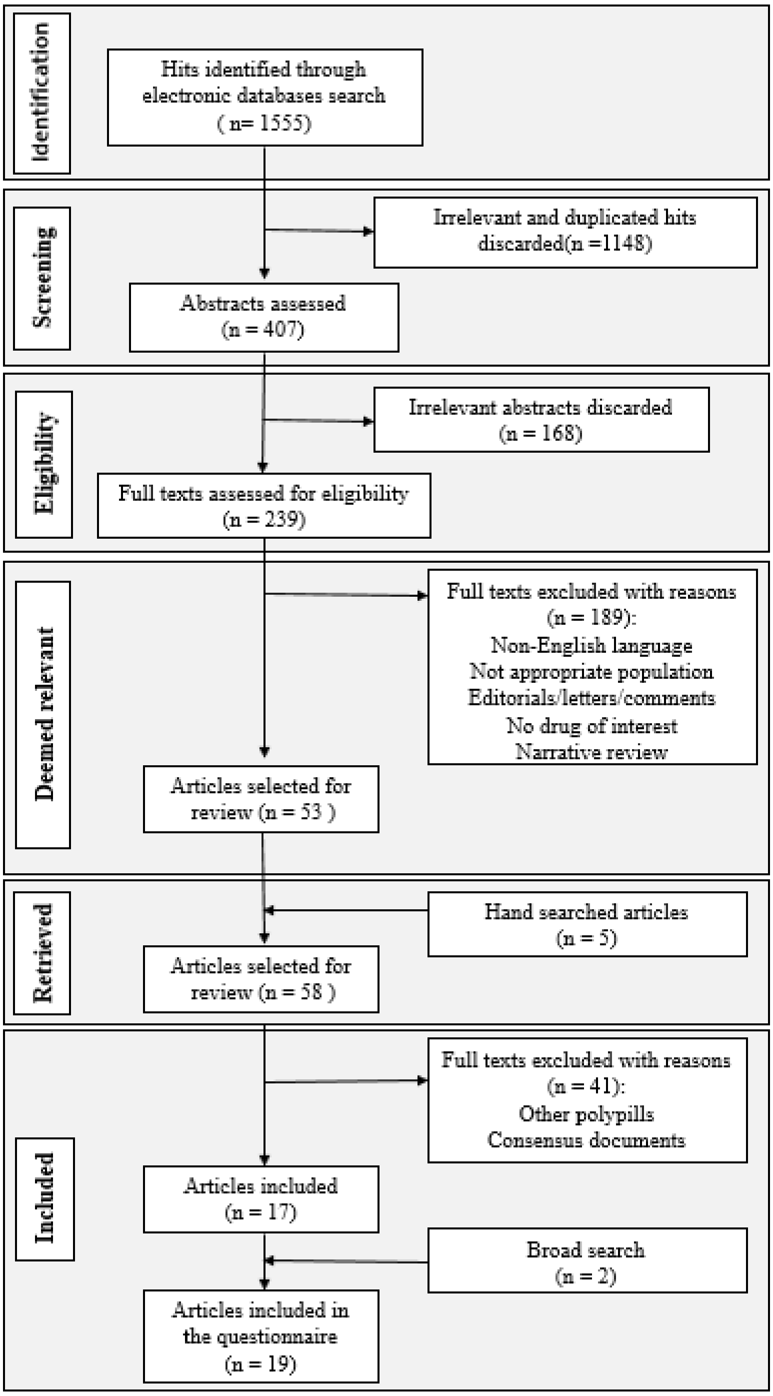

Articles identified in broad search: (32,38)

Figure S2. Example of a revised statement assessed in Delphi consultation round 2

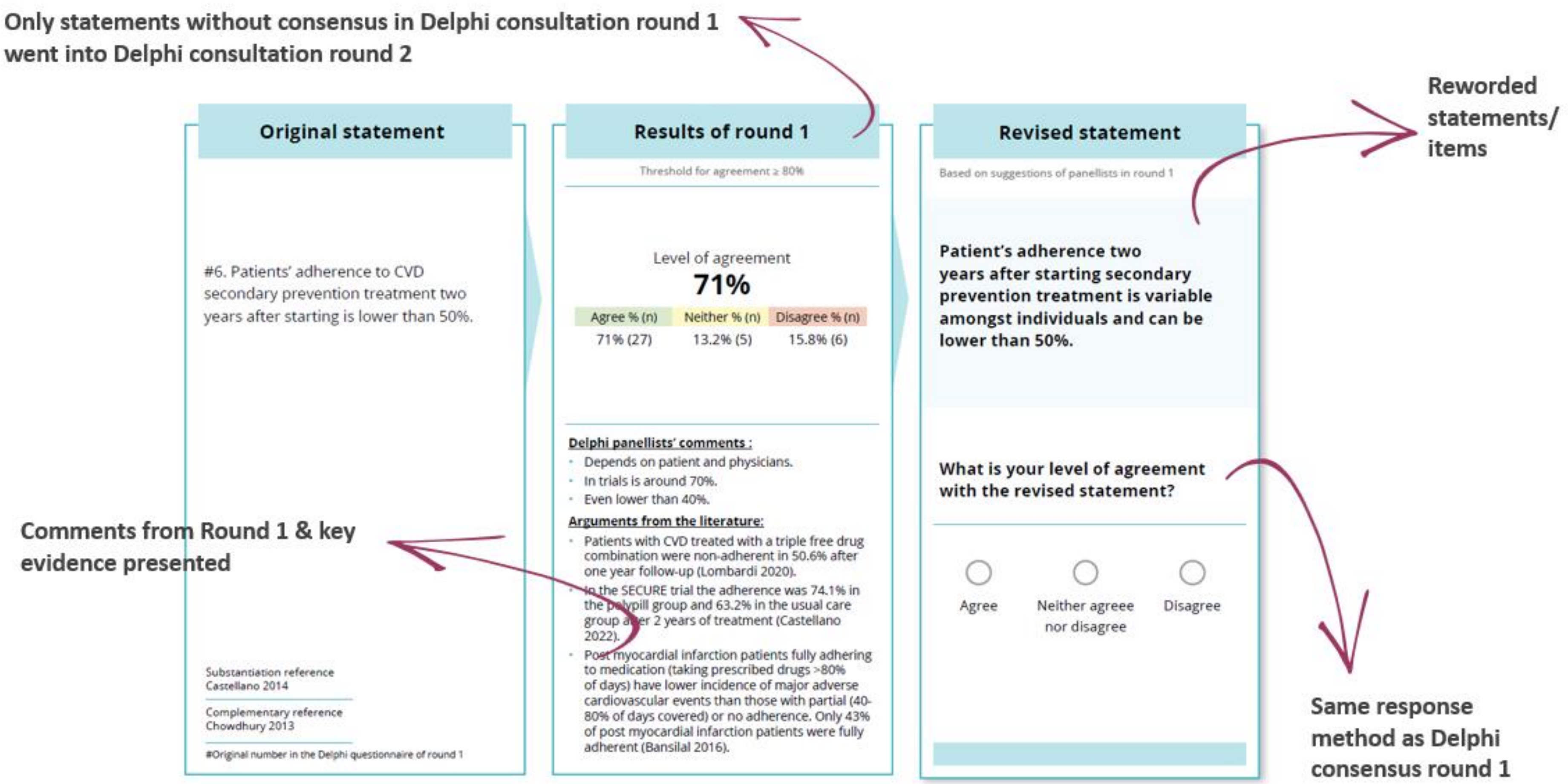

Figure S3. Consensus statements flow

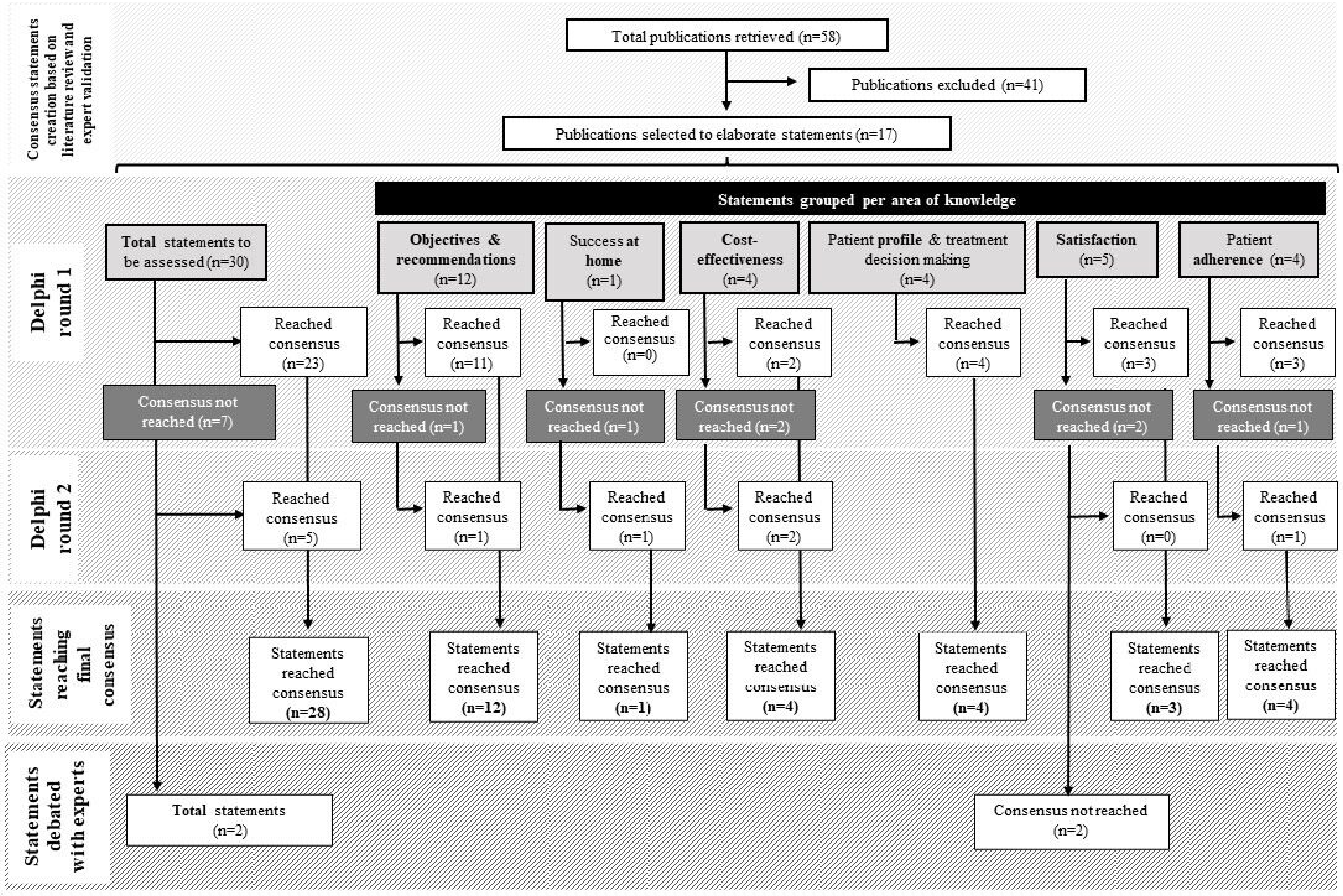

Figure S4. Percentage of Delphi panellists agreeing on the use of the cardiovascular polypill* in patients with various clinical profiles

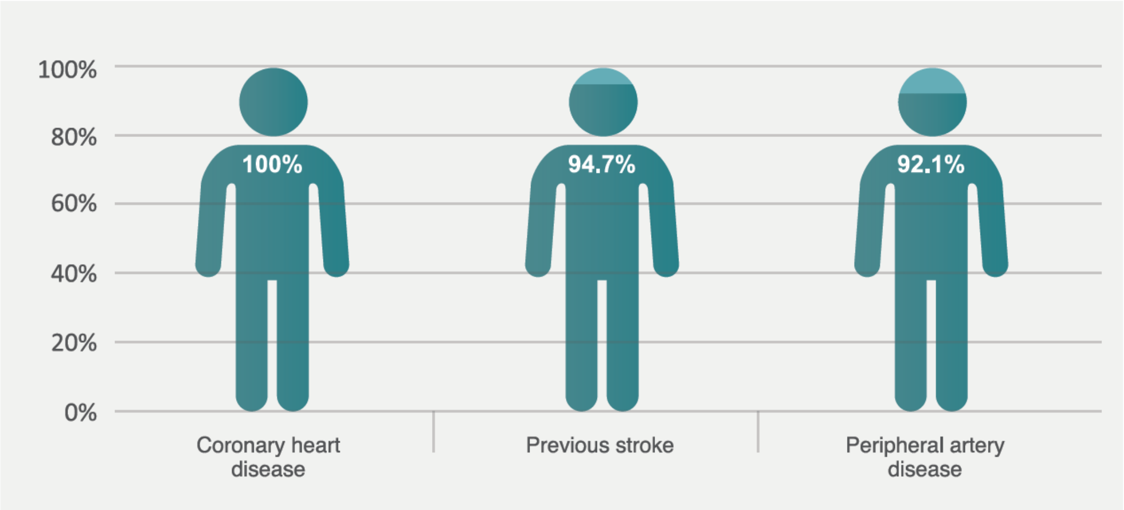

***Cardiovascular polypill developed by the Centro Nacional de Investigaciones Cardiovascualres (CNIC), also known as CNIC-polypill. This polypill contains acetylsalicylic acid (100mg), atorvastatin (20 or 40mg), and ramipril (2.5, 5, or 10mg) and it is available in 23 countries as Trinomia®, Sincronium® or Iltria®.**

Figure S5. Percentage of Delphi panellists agreeing on the importance of factors related to decisions about using the cardiovascular polypill* in CHD patients

A. Sociodemographic characteristics

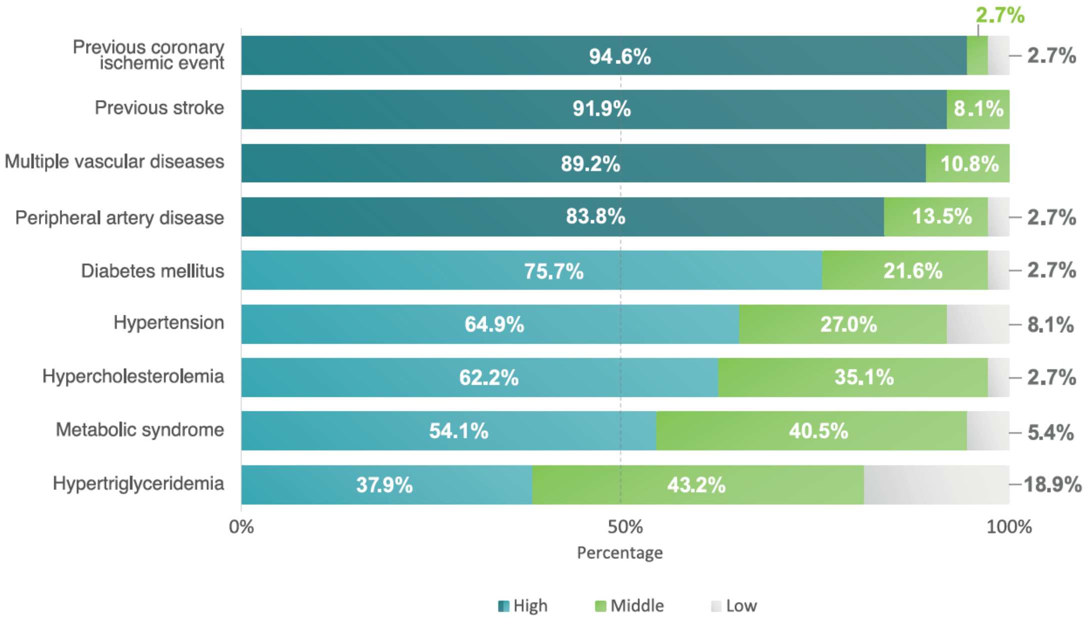

B. Clinical characteristics

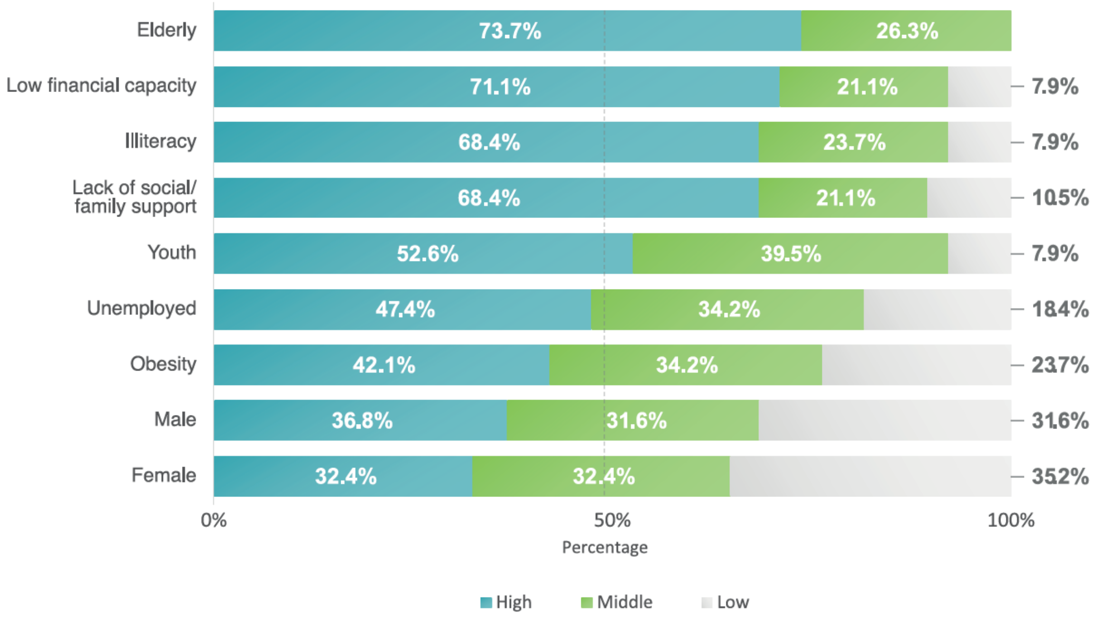

C. Treatment history

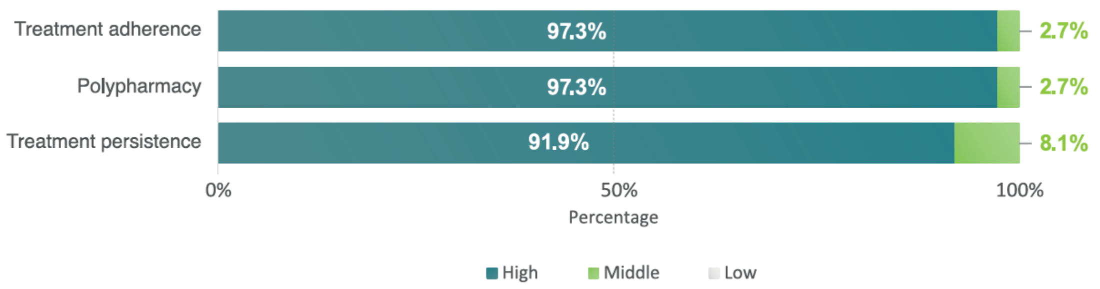

* Cardiovascular polypill developed by the Centro Nacional de Investigaciones Cardiovascualres (CNIC), also known as CNIC-polypill. This polypill contains acetylsalicylic acid (100mg), atorvastatin (20 or 40mg), and ramipril (2.5, 5, or 10mg) and it is available in 23 countries as Trinomia®, Sincronium® or Iltria®.

Statements rated as highly important by ≥80% of panellists (the consensus threshold) are highlighted in intense green.

CHD: coronary heart disease

References

1. Jünger S, Payne SA, Brine J, Radbruch L, Brearley SG. Guidance on Conducting and REporting DElphi Studies (CREDES) in palliative care: Recommendations based on a methodological systematic review. Palliat Med. 2017 Sep 1;31(8):684–706.

2. Beiderbeck D, Frevel N, von der Gracht HA, Schmidt SL, Schweitzer VM. Preparing, conducting, and analyzing Delphi surveys: Cross-disciplinary practices, new directions, and advancements. MethodsX [Internet]. 2021;8:101401. Available from: https://doi.org/10.1016/j.mex.2021.101401

3. Hasson F, Keeney S, McKenna H. Research guidelines for the Delphi survey technique. J Adv Nurs. 2000;32(4):1008–15.

4. Barrett D, Heale R. What are Delphi studies? Evid Based Nurs. 2020;23(3):68–9.

5. Steinmann G, Delnoij D, Van De Bovenkamp H, Groote R, Ahaus K. Expert consensus on moving towards a value-based healthcare system in the Netherlands: A Delphi study. BMJ Open. 2021;11(4).

6. Minkman M, Ahaus K, Fabbricotti I, Nabitz U, Huijsman R. A quality management model for integrated care: results of a Delphi and Concept Mapping study. Int J Qual Heal care J Int Soc Qual Heal Care. 2009 Feb;21(1):66–75.

7. Mora Pinzon MC, Myers S, Renken J, Eggert E, Chewning B, Mahoney JE. Essential elements to 'design for dissemination' within a research network-a modified Delphi study of the Community-Academic Aging Research Network (CAARN). Implement Sci Commun. 2021 Feb;2(1):18.

8. Shah HA, Kalaian SA. Which is the best parametric statistical method for analyzing delphi data? J Mod Appl Stat Methods. 2009;8(1):226–32.

9. Rodríguez-Mañas L, Féart C, Mann G, Viña J, Chatterji S, Chodzko-Zajko W, et al. Searching for an operational definition of frailty: a Delphi method based consensus statement: the frailty operative definition-consensus conference project. J Gerontol A Biol Sci Med Sci. 2013 Jan;68(1):62–7.

10. Richards KL, Woolrych I, Allen KL, Schmidt U. A Delphi study to explore clinician and lived experience perspectives on setting priorities in eating disorder services. BMC Health Serv Res. 2022 Jun;22(1):788.

11. Taylor E. We Agree, Don't We? The Delphi Method for Health Environments Research. Heal Environ Res Des J. 2020;13(1):11–23.

12. Chyung SYY, Roberts K, Swanson I, Hankinson A. Evidence-Based Survey Design: The Use of a Midpoint on the Likert Scale. Perform Improv. 2017;56(10):15–23.

13. Khadka J, Gothwal VK, McAlinden C, Lamoureux EL, Pesudovs K. The importance of rating scales in measuring patient-reported outcomes. Health Qual Life Outcomes. 2012;10:1–13.

14. Gattrell WT, Logullo P, van Zuuren EJ, Price A, Hughes EL, Blazey P, et al. ACCORD (ACcurate COnsensus Reporting Document): A reporting guideline for consensus methods in biomedicine developed via a modified Delphi. PLoS Med [Internet]. 2024;21(1):1–18. Available from: http://dx.doi.org/10.1371/journal.pmed.1004326

15. Wald NJ, Law MR. A strategy to reduce cardiovascular disease by more than 80%. BMJ. 2003 Jun 28;326(7404):1419–23.

16. Lombardi N, Crescioli G, Simonetti M, Marconi E, Vannacci A, Bettiol A, et al. Adherence to Triple-Free-Drug Combination Therapies Among Patients With

Cardiovascular Disease. Am J Cardiol. 2020 May;125(9):1429–35.

17. Castellano JM, Pocock SJ, Bhatt DL, Quesada AJ, Owen R, Fernandez-Ortiz A, et al. Polypill Strategy in Secondary Cardiovascular Prevention. N Engl J Med. 2022 Sep 15;387(11):967–77.

18. Bansilal S, Castellano JM, Garrido E, Wei HG, Freeman A, Spettell C, et al. Assessing the Impact of Medication Adherence on Long-Term Cardiovascular Outcomes. J Am Coll Cardiol. 2016 Aug;68(8):789–801.

19. Cosin-Sales J, Murcia-Zaragoza JM, Pereyra-Rico HO, La Guía-Galipienso F De, Hermans K, Rubio G. Evaluating patients' satisfaction and preferences with a secondary prevention cardiovascular polypill: the Aurora Study. J Comp Eff Res. 2021 Sep 1;10(13):975–85.

20. Rohatgi KW, Humble S, McQueen A, Hunleth JM, Chang S-H, Herrick CJ, et al. Medication Adherence and Characteristics of Patients Who Spend Less on Basic Needs to Afford Medications. J Am Board Fam Med. 2021;34(3):561–70.

21. Wilke T, Weisser B, Predel HG, Schmieder RE, Wassmann S, Gillessen A, et al. Effects of cardiovascular single pill combinations compared with identical multi-pill therapies on healthcare cost and utilization in Germany. J Comp Eff Res. 2022;11(6):411–22.

22. Cordero A, Dalmau González-Gallarza R, Masana L, Fuster V, Castellano JM, Ruiz Olivar JE, et al. Economic Burden Associated with the Treatment with a Cardiovascular Polypill in Secondary Prevention in Spain: Cost-Effectiveness Results of the NEPTUNO Study. Clinicoecon Outcomes Res. 2023;15:559–71.

23. Becerra V, Gracia A, Desai K, Abogunrin S, Brand S, Chapman R, et al. Cost-effectiveness and public health benefit of secondary cardiovascular disease prevention from improved adherence using a polypill in the UK. BMJ Open. 2015;5(5).

24. Barrios V, Kaskens L, Castellano JM, Cosin-Sales J, Ruiz JE, Zsolt I, et al. Usefulness of a Cardiovascular Polypill in the Treatment of Secondary Prevention Patients in Spain: A Cost-effectiveness Study. Rev Esp Cardiol (Engl Ed). 2017 Jan;70(1):42–9.

25. Aguiar C, Araujo F, Rubio-Mercade G, Carcedo D, Paz S, Castellano JM, et al. Cost-Effectiveness of the CNIC-Polypill Strategy Compared With Separate Monocomponents in Secondary Prevention of Cardiovascular and Cerebrovascular Disease in Portugal: The MERCURY Study. J Heal Econ Outcomes Res. 2022;9(2):134–46.

26. Ros-Castelló V, Natera-Villalba E, Gómez-López A, Sánchez-Sánchez A, Chico-García JL, García-Madrona S, et al. Use of the Cardiovascular Polypill in Secondary Prevention of Cerebrovascular Disease: A Real-Life Tertiary Hospital Cohort Study of 104 Patients. Cerebrovasc Dis Extra. 2020 Sep 1;10(3):166–73.

27. González-Juanatey JR, Cordero A, Castellano JM, Masana L, Dalmau R, Ruiz E, et al. The CNIC-Polypill reduces recurrent major cardiovascular events in real-life secondary prevention patients in Spain: The NEPTUNO study. Int J Cardiol. 2022 Aug;361:116–23.

28. Araújo F, Caldeira D, Aguiar C, Antunes JP, Cardim N, Cunha V, et al. Polypill use for the prevention of cardiovascular disease: a position paper. Rev Port Cardiol. 2023 May;(10):861–72.

29. Grigorian-Shamagian L, Edel K, Esteve-Pastor MA, Aceña Á, Silva C, Delgado-Silva J, et al. Practical Decision Algorithms for the Use of the Cardiovascular Polypill in Secondary Prevention in Europe. Front Cardiovasc Med. 2021 Aug 24;8:663361.

30. Visseren FLJ, MacH F, Smulders YM, Carballo D, Koskinas KC, Bäck M, et al. 2021

ESC Guidelines on cardiovascular disease prevention in clinical practice. Eur Heart J. 2021 Sep 7;42(34):3227–337.

31. Coca A, Kreutz R, Manolis AJ, Mancia G. A practical approach to switch from a multiple pill therapeutic strategy to a polypill-based strategy for cardiovascular prevention in patients with hypertension. J Hypertens. 2020 Oct 1;38(10):1890–8.

32. Agency of Healthcare Research and Quality. Strategy 4: Ideal Discharge Planning (Implementation Handbook) Guide to Patient and Family Engagement Care Transitions from Hospital to Home [Internet]. 2017. [cited 2022 Oct 11]. Available from: https://www.ahrq.gov/sites/default/files/wysiwyg/professionals/systems/hospital/engagingfamilies/strategy4/Strat4_Implement_Hndbook_508_v2.pdf

33. Marquina C, Zomer E, Vargas-Torres S, Zoungas S, Ofori-Asenso R, Liew D, et al. Novel Treatment Strategies for Secondary Prevention of Cardiovascular Disease: A Systematic Review of Cost-Effectiveness. Pharmacoeconomics. 2020;38(10):1095–113.

34. Jahangiri R, Rezapour A, Malekzadeh R, Olyaeemanesh A, Roshandel G, Motevalian SA. Cost-effectiveness of fixed-dose combination pill (Polypill) in primary and secondary prevention of cardiovascular disease: A systematic literature review. PLoS One. 2022 Jul 1;17(7):e0271908.

35. Virdee SK, Greenfield SM, Fletcher K, McManus RJ, Hobbs FDR, Mant J. Would primary healthcare professionals prescribe a polypill to manage cardiovascular risk? A qualitative interview study. BMJ Open. 2013 Jan 1;3(3):e002498.

36. Murphy A, Willis R, Ansbro É, Masri S, Kabbara N, Dabbousy T, et al. Implementation of fixed-dose combination therapy for secondary prevention of atherosclerotic cardiovascular disease among Syrian refugees in Lebanon: a qualitative evaluation. BMC Health Serv Res. 2022 Dec 1;22(1):1–12.

37. Castellano JM, Sanz G, Peñalvo JL, Bansilal S, Fernández-Ortiz A, Alvarez L, et al. A polypill strategy to improve adherence: results from the FOCUS project. J Am Coll Cardiol. 2014;64(20):2071–82.

38. Mortelmans L, De Baetselier E, Goossens E, Dilles T. What Happens after Hospital Discharge? Deficiencies in Medication Management Encountered by Geriatric Patients with Polypharmacy. Int J Environ Res Public Health. 2021 Jun 30;18(13):7031.
